# Longitudinal Patient-Reported Outcome Trajectories in Long COVID: Findings From the STOP-PASC Clinical Trial

**DOI:** 10.1093/ofid/ofaf634

**Published:** 2025-10-08

**Authors:** Prasanna Jagannathan, Haley Hedlin, Jane W Liang, Blake Shaw, Evan Maestri, Michelle Lin, P J Utz, Upinder Singh, Linda N Geng, Hector Bonilla

**Affiliations:** Department of Medicine, Stanford University School of Medicine, Stanford, California, USA; Stanford Quantitative Sciences Unit, Stanford, California, USA; Stanford Quantitative Sciences Unit, Stanford, California, USA; Division of Research, Kaiser Permanente Northern California, Pleasanton, California, USA; Stanford Quantitative Sciences Unit, Stanford, California, USA; Department of Medicine, Stanford University School of Medicine, Stanford, California, USA; Stanford Quantitative Sciences Unit, Stanford, California, USA; Department of Medicine, Stanford University School of Medicine, Stanford, California, USA; Department of Medicine, Stanford University School of Medicine, Stanford, California, USA; Department of Internal Medicine, University of Iowa Carver College of Medicine, Iowa City, Iowa, USA; Department of Medicine, Stanford University School of Medicine, Stanford, California, USA; Department of Medicine, Stanford University School of Medicine, Stanford, California, USA

**Keywords:** Long COVID, Paxlovid, Trajectory mapping

## Abstract

**Background:**

Long COVID is a heterogeneous post-infectious condition. Although patient-reported outcome (PRO) measures for diagnosis or therapeutic monitoring have been adapted from related complex chronic illnesses, no PRO has been validated specifically in Long COVID. The STOP-PASC randomized, placebo-controlled trial of nirmatrelvir/ritonavir (NMV/r) in adults with Long COVID showed no overall treatment effect. This exploratory analysis aimed to identify distinct symptom trajectories and clinical characteristics associated with improvement or worsening over time.

**Methods:**

We performed latent class trajectory modeling (LCTM) on PRO measures—including the Patient Global Impression of Severity (PGIS), Patient Global Impression of Change (PGIC), PROMIS domains, and core symptoms—among 155 randomized participants. Participants were followed for 15 weeks with serial symptom assessments. Trajectory groups were identified using Bayesian Information Criteria and characterized using descriptive statistics and absolute standardized differences.

**Results:**

LCTM revealed heterogeneity in symptom trajectories. Two groups emerged for PGIS (improving *n* = 17, persistent/severe *n* = 136) and PGIC (improving *n* = 130; worsening *n* = 22). PROMIS-Physical Function modeling identified four groups (improving, normal/mild, moderate, and severe), fatigue core symptom modeling identified three (improving; moderate; severe). Worsening groups had higher proportions of NMV/r-treated participants and greater prevalence of cardiovascular symptoms and low-dose naltrexone use. Improving groups had shorter time since infection and higher baseline physical function. No subgroup showed a clear benefit from NMV/r.

**Conclusions:**

Distinct PRO trajectories reflect the clinical heterogeneity of Long COVID. NMV/r showed no clear benefit across subgroups. These findings emphasize the need for validated, Long COVID-specific PRO instruments and targeted therapeutic trials tailored to Long COVID subtypes.

Long COVID is an infection-associated chronic condition that occurs after SARS-CoV-2 infection and is present for at least 3 months as a continuous, relapsing and remitting, or progressive disease state that affects one or more organ systems [1, 2]. Long COVID remains a major public health problem in the wake of the pandemic, with recent estimates suggesting that about 8.4% of US adults have experienced Long COVID and about 3.6% currently have Long COVID [3]. Long COVID encompasses various manifestations and symptoms, such as fatigue, brain fog, and dyspnea that can significantly impact quality of life and function. Several survey instruments have been proposed in clinical and research settings to measure Long COVID-associated symptoms based on patient-reported outcomes (PROs, reviewed in [4, 5]). This includes Likert-based assessment of individual symptoms linked to Long COVID [6]; the World Health Organization Post COVID-19 Case Record Form [7]; Patient Global Impression of Change (PGIC) and Patient Global Impression of Severity (PGIS) scales; and the National Institutes of Health (NIH) Patient-Reported Outcomes Measurement Information System (PROMIS) standardized item banks to measure PROs relevant across common medical conditions [8, 9]. The PGIC and PROMIS surveys have been used in the NIH-funded RECOVER trials (https://trials.recoverCOVID.org) and other Long COVID trials [9]. However, none of these instruments were originally designed for, nor have been validated in, Long COVID. Existing PRO instruments may be suboptimal for Long COVID's unique characteristics, including their limited sensitivity to symptom fluctuation and lack of specificity for the multi-system manifestations that define this condition. The lack of validated PROs for Long COVID hinders the development of biomarkers to assist with diagnosis or measures to mark the response to putative treatments, whether repurposed antivirals, anti-inflammatory agents, or symptomatic therapies.

We recently conducted a randomized blinded placebo-controlled trial to investigate the efficacy and safety of a 15-day course of nirmatrelvir/ritonavir (PAXLOVID) versus placebo/ritonavir in participants with Long COVID to test the hypothesis that SARS-CoV-2 virus or viral particle persistence may be a causal mechanism for Long COVID (STOP-PASC trial) [6]. The primary outcome was a pooled severity of 6 core symptoms (fatigue, brain fog, dyspnea, body aches (including muscle and/or joint pain), gastrointestinal symptoms (including nausea, vomiting, diarrhea, constipation, abdominal pain, or decreased appetite) and cardiovascular symptoms (including chest pain, fast heart rate, palpitations, or lightheadedness), and secondary outcomes included PGIC, PGIS, and PROMIS measures (physical function, cognitive function, fatigue, and dyspnea). Overall, we found no clear differences between the intervention arms in primary and secondary outcomes. However, there was significant individual heterogeneity among study participants, and it was unclear whether there were specific subgroups who may have had differing trajectories and outcomes from the overall cohort.

Given the heterogeneity of Long COVID, this exploratory analysis aimed to address two key objectives. First, we aimed to identify distinct subgroups of individuals with Long COVID within the clinical trial cohort using latent class trajectory modeling (LCTM). Second, we utilized these groupings to explore features that distinguish individuals with improving symptoms from those with persistent or worsening symptoms. Utilizing this approach, we hoped to uncover potential subpopulations of individuals that may have responded to nirmatrelvir/ritonavir and/or have other notable features that could inform diagnostic/therapeutic development. This approach not only sheds light on the heterogeneity of Long COVID but also seeks to inform endpoint selection, the development of targeted interventions for future therapeutic studies, and comparisons with recent and ongoing trials.

## METHODS

### Study Population

This is an exploratory analysis of data from participants enrolled in a single-center, randomized, blinded, placebo-controlled pilot trial to evaluate the efficacy and safety of nirmatrelvir/ritonavir (PAXLOVID) in treating Long COVID in adults. One hundred and fifty-five participants with Long COVID who met all the inclusion criteria were randomized 2:1 to a 15-day course of twice-daily (a) nirmatrelvir/ritonavir (nirmatrelvir 300 mg–ritonavir 100 mg; NMV/*r*) or (b) placebo/ritonavir (placebo 0 mg–ritonavir 100 mg; PBO/r). A 2:1 randomization was selected for recruitment purposes based on patient/community advisory input and to help detect safety events. A total of 102 and 53 participants were enrolled in the two arms above, respectively. For each participant, the study lasted 15 weeks (3.5 months). Symptoms severity assessments, patient-reported outcomes (PROs), and clinical assessments were performed at each time point. The rationale for selection of primary and secondary outcome measures for this trial was provided in the main paper [6]. All participants in the STOP-PASC study were included in this exploratory analysis.

### Patient Consent Statement

This study was approved by the Stanford Institutional Review Board. All participants gave written informed consent.

### Outcome Measures

The two primary outcome measures studied in this exploratory analysis include (1) PGIS at baseline, 15 days, 5 weeks, 10 weeks, and 15 weeks; and (2) PGIC at 15 days, 5 weeks, 10 weeks, and 15 weeks. Secondary outcome measures studied included PROMIS Physical Function (PF) SF 4a v2.0 T-score at baseline, 15 days, 5 weeks, 10 weeks, and 15 weeks; PROMIS Fatigue SF 7a v1.0 T-score at baseline, 15 days, 5 weeks, 10 weeks, and 15 weeks; PROMIS Dyspnea-Severity SF 5a v1.0 T-score at baseline, 15 days, 5 weeks, 10 weeks, and 15 weeks; PROMIS Cognitive Function (CF) Abilities T-score at baseline, 15 days, 5 weeks, 10 weeks, and 15 weeks; and core symptom severity scores at each weekly timepoint (biweekly after 10 weeks). Core symptoms included: fatigue, brain fog, dyspnea, body aches (including muscle and/or joint pain), gastrointestinal symptoms (including nausea, vomiting, diarrhea, constipation, abdominal pain, or decreased appetite), and cardiovascular symptoms (including chest pain, fast heart rate, palpitations, or lightheadedness). The severity of core symptoms was assessed on a 4-point Likert scale (0 = none, 1 = mild, 2 = moderate, and 3 = severe) with participants rating their symptom burden at its worst during the past 7 days.

### Statistical Analysis

Eligible participants' baseline characteristics were summarized and compared between the two treatment arms.

Using the lcmm [10, 11] and LCTMtools [12, 13] R packages, we performed latent class trajectory modeling of patient-reported outcome measures to identify groups. We opted to use LCTM instead of alternative clustering approaches because LCTMs explicitly model longitudinal trajectories. Derived from mixed models, LCTMs readily handle missing data under missing-at-random (MAR) assumptions and estimate class probabilities. Many clustering approaches require complete case data and/or return only class labels. Trajectories were modeled using each longitudinal outcome (PGIC, PGIS, PROMIS measures, and core symptom severity; Supplementary Methods). We assessed different random effect structures and number of groups using the Bayesian information criteria (BIC) to arrive at a final best-fit model for each outcome (Supplementary Table 1). In the case of ties, the simpler model was favored. Note that this data-driven approach does not guarantee that the final model for a given outcome trajectory will separate patients into distinct groups, ie, it is possible for the “best” model to have only one group. For best-fit models that identified more than one group, we also report group-specific diagnostics (Supplementary Table 2). Group stability for each final model was quantified using cluster consensus statistics obtained from the resampling algorithm outlined by Monti et al [14].

No additional covariates were included in the models. For each best-fit model that identified more than one group, we visualized the cluster consensus indices (Supplementary Figures 1–8).

Our analysis included all randomized participants, including those who did not provide any follow-up data. Missing observations were accounted for by the mixed model framework in the LCTM approach. LCTMs use maximum likelihood estimation to straightforwardly handle missing data under missing at random (MAR) assumptions without the need for multiple imputation. Participants with only baseline values for a given outcome were still included when model-fitting (contributing only their baseline measurements), but the models were unable to classify them because they did not have outcome trajectories. No patients were missing baseline measurements.

After groups were identified, patient trajectories within each group were visualized using heatmaps and alluvial plots. The identified groups were characterized using descriptive statistics of demographics and other baseline measures. As this was an exploratory analysis, no *a priori* hypothesis testing was planned. Instead, we used absolute standardized differences (ASDs) to evaluate the magnitude of differences between groups. A larger ASD indicates a larger difference between the groups (eg, 0.2 = small difference, 0.5 = moderate difference, 0.8 = large difference).

As a secondary analysis, cross-tabulation was used to examine the overlap between groups defined using the primary outcomes as well as the correlation between PGIC at week 10 and changes in PGIS measurements measured between week 5 and week 10. In a *post hoc* analysis, we also created a heatmap, alluvial plot, and descriptive statistics showing the groups defined by the PGIC and PGIS groups identified from LCTM.

## RESULTS

### Study Population

One hundred and fifty-five participants were enrolled in the STOP-PASC study, of which 153 had at least one follow-up observation, comprising 102 NMV/r participants and 53 PBO/r participants. Baseline demographics were previously reported [6] (Supplementary Table 3). More than 90% of participants reported a PGIS score of moderate or greater at enrollment. The most bothersome symptom at enrollment was fatigue (45% of participants), with brain fog (24.5%) being the next most common symptom reported. The mean duration between the index infection and randomization was 536 days (SD = 275 days, Supplementary Table 3).

### Trajectory Modeling of PGIS Scores Identifies Two Participant Groups

**Figure 1.**
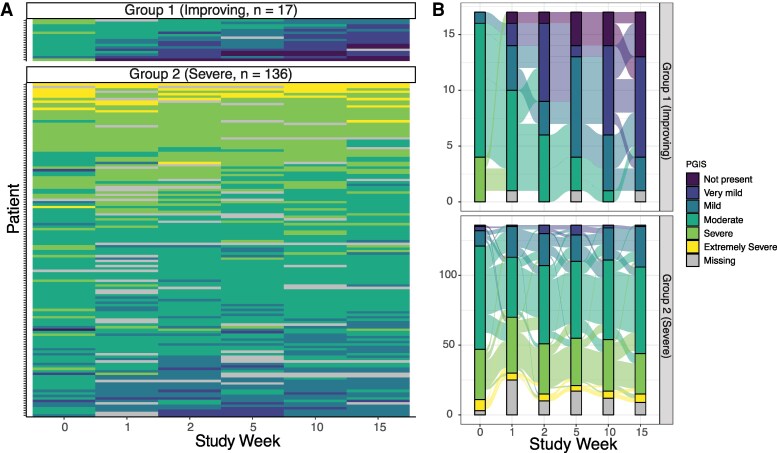
PGIS trajectories visualized within modeling groups using heatmaps (*A*) and alluvial plots (*B*). Darker colors indicate better PGIS scores and lighter colors indicate worse scores.

We utilized latent class trajectory modeling as a data driven approach to identify different Long COVID patient-related outcome trajectories following enrollment, first focusing on global PRO measures. For PGIS, which asked, “Please rate the severity of your Long COVID symptoms right now”, modeling identified two trajectory groups: one group with what appears to be improving PGIS scores over time (*N* = 17), and the other with what appears to be stable, moderate to severe PGIS trajectories (*n* = 136, Figure 1).

Female participants comprised a higher proportion of individuals in the severe PGIS group (61.0%) compared with the improving group (47.1%, ASD = 0.28, Table 1). The proportion of participants receiving NMV/r was similar in both severe versus improving PGIS groups (64.7 vs 66.2%). The mean duration between the index infection and randomization was shorter in the improving group compared with the worsening group (373 days vs 555 days, ASD = 0.78). At the time of enrollment, one minute sit-to-stand repetitions were higher in the improving (26.9) versus severe (19.5) groups (ASD = 0.58). More participants in the severe group reported that cardiovascular symptoms were their most bothersome symptom at baseline (16.2% vs 0% in improving group). Low dose naltrexone was more commonly being taken at baseline among individuals in the severe (20%) versus improving (5.9%, ASD = 0.43) group. Other concomitant medications at baseline were similar between PGIS groups (Supplementary Table 4).

**Table 1.** Characterizing PGIS Longitudinal Groups

Characteristics	Group 1 (Improving) (*n* = 17)	Group 2 (Severe) (*N* = 136)	ASD
NMV/r arm (%)	11 (64.7)	90 (66.2)	0.03
Female (%)	8 (47.1)	83 (61.0)	0.28
Age (mean (SD))	44.65 (16.05)	44.09 (13.03)	0.04
Race (%)			0.41
American Indian/Alaska Native	0 (0.0)	0 (0.0)	
Asian	2 (11.8)	18 (13.2)	
Native Hawaiian or other Pacific Islander	0 (0.0)	1 (0.7)	
Black or African American	0 (0.0)	3 (2.2)	
White	14 (82.4)	99 (72.8)	
More than one race	0 (0.0)	6 (4.4)	
Unknown	1 (5.9)	9 (6.6)	
Most bothersome symptom (%)			0.86
Fatigue	9 (52.9)	60 (44.1)	
Shortness of breath	0 (0.0)	4 (2.9)	
Brain fog	7 (41.2)	31 (22.8)	
Body aches	1 (5.9)	12 (8.8)	
Cardiovascular symptoms	0 (0.0)	22 (16.2)	
Gastrointestinal symptoms	0 (0.0)	7 (5.1)	
Heart rate (supine to standing) (mean (SD))	8.53 (8.68)	6.39 (11.04)	0.22
1 min sit-to-stand (mean (SD))	26.88 (15.16)	19.54 (9.35)	0.58
Days from index infection to baseline (mean (SD))	372.82 (177.55)	554.66 (280.20)	0.78
Days from most recent infection to baseline (mean (SD))	312.12 (166.48)	423.46 (283.84)	0.480

ASDs (absolute standardized differences) are reported to assess the magnitude of differences between groups: a larger ASD indicates a larger difference between the groups (eg, 0.2 = small difference, 0.5 = moderate difference, 0.8 = large difference).

### Trajectory Modeling Identifies Two Participant Groups of Patient Global Impression of Change (PGIC)

**Figure 2.**
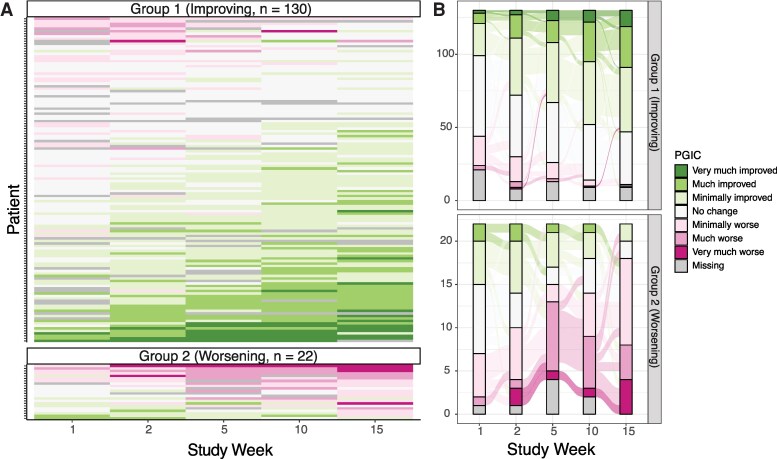
PGIC trajectories visualized within modeling groups using heatmaps (*A*) and alluvial plots (*B*).

For PGIC, which asks, “Since the start of the study, my overall status is …”, modeling identified two trajectory groups: one group with what appears to be improving PGIC scores over time (*N* = 130), and the other with what appears to be worsening PGIC trajectories (*n* = 22, Figure 2).

Female participants comprised a higher proportion of individuals in the improving PGIC group (63.8%) compared with the worsening group (31.8%, ASD = 0.68, Table 2). The improving group was comprised of a lower proportion of participants receiving NMV/r (63.8%) compared with the worsening group (81.8%). The mean duration between the index infection and randomization was shorter in the improving group compared with the worsening group (517 days vs 625 days, ASD = 0.39). A slightly higher proportion of participants in the worsening cluster reported that cardiovascular symptoms were their most bothersome symptom at baseline (18.2% vs 13.8% in improving group). Concomitant medications at baseline were similar between those in the improving versus worsening PGIC groups (Supplementary Table 5).

**Table 2.** Characterizing PGIC Longitudinal Groups

Baseline Characteristics	Group 1 (Improving) (*n* = 130)	Group 2 (Worsening) (*n* = 22)	ASD
NMV/r arm (%)	83 (63.8)	18 (81.8)	0.41
Female (%)	83 (63.8)	7 (31.8)	0.68
Age (mean (SD))	43.74 (13.34)	46.27 (13.66)	0.19
Race (%)			0.47
Asian	19 (14.6)	1 (4.5)	
Black or African American	3 (2.3)	0 0.0)	
White	93 (71.5)	19 (86.4)	
More than one race	5 (3.8)	1 (4.5)	
Other/Unknown	10 (7.7)	1 (4.5)	
Most bothersome symptom (%)			0.39
Fatigue	60 (46.2)	9 (40.9)	
Shortness of breath	3 (2.3)	1 (4.5)	
Brain fog	31 (23.8)	6 (27.3)	
Body aches	11 (8.5)	2 (9.1)	
Cardiovascular symptoms	18 (13.8)	4 (18.2)	
Gastrointestinal symptoms	7 (5.4)	0 (0.0)	
Heart rate (supine to standing) (mean (SD))	6.38 (10.98)	8.55 (9.70)	0.21
1 min sit-to-stand (mean (SD))	20.41 (10.50)	20.36 (9.75)	<0.01
Days from index infection to baseline (mean (SD))	516.96 (272.82)	625.00 (284.66)	0.39
Days from most recent infection to baseline (mean (SD))	401.41 (265.23)	449.86 (325.29)	0.16

ASDs (absolute standardized differences) are reported to assess the magnitude of differences between groups: a larger ASD indicates a larger difference between the groups (eg, 0.2 = small difference, 0.5 = moderate difference, 0.8 = large difference).

As PGIC is a measure of change from baseline, and PGIS is a measure of severity at a given visit, we sought to determine whether PGIC at week 10 correlated with changes in PGIS measurements measured at week 5 and week 10. 60/72 (83%) of participants who reported that their symptoms either had not changed or minimally changed by PGIC had no change in their PGIS score between weeks 5 and 10, confirming robust correlation between these measures (Supplementary Table 6). Furthermore, all 22 participants identified in the worsening PGIC group also clustered in the severe PGIS group, and all 17 participants in the improving PGIS cluster were also identified as improving by PGIC (Supplementary Table 7). A *post hoc* analyses of characteristics of groups defined by the combination of PGIC and PGIS clusters were similar to the individual comparisons and provided in the supplement (Supplementary Table 8).

### Trajectory Modeling Identifies Three Participant Groups of PROMIS-Physical Function

**Figure 3.**
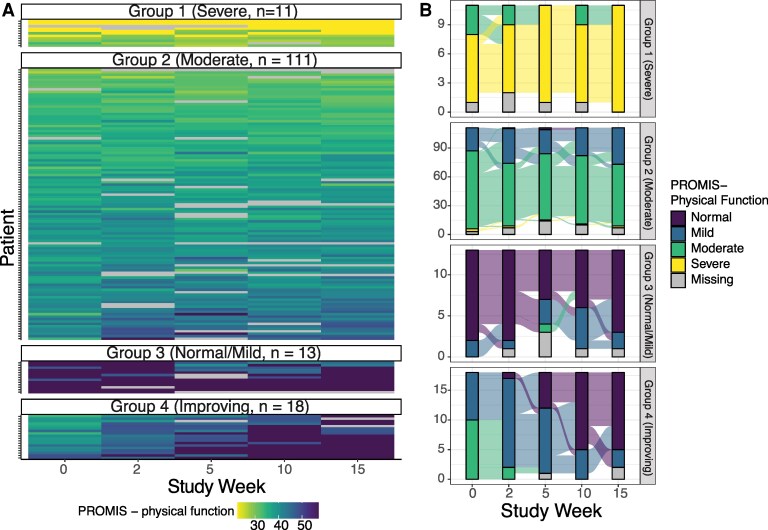
PROMIS-physical function trajectories visualized within modeling groups using heatmaps (*A*) and alluvial plots (*B*).

For PROMIS measures, trajectory modeling did not distinguish multiple groups for the Fatigue, Dyspnea, and CF measures. For PROMIS-PF, four trajectories were identified: one group with what appears to have improving PROMIS-PF scores (*n* = 18), one with what appears to have stable and normal/mild PROMIS-PF scores over time (*N* = 13), one with what appears to be stable but moderate PROMIS-PF scores over time (*n* = 111), and one with what appears to be stable and severe PROMIS-PF scores over time (*n* = 11, Figure 3).

Female participants comprised a higher proportion of individuals in the moderate (63.1%) and severe PROMIS-PF group (72.7%) compared with those in the good (30.8%) or improving (50.8%) groups (ASD = 0.494, Supplementary Table 9). The proportion of participants receiving NMV/r was similar in the severe (63.6%), moderate (69.4%), and improving groups (66.7%) but lower in the normal/mild groups (38.5%). The mean duration between the index infection and randomization was shorter in the improving group (374 days) compared with the normal/mild (507 days), moderate (571 days), and severe (464 days) groups (ASD = 0.422). At the time of enrollment, 1 min sit-to-stand repetitions were lower in the severe group (10.6) versus the moderate (20), normal/mild (26), and improving (25) groups. More participants in the severe PROMIS-PF group reported that cardiovascular symptoms were their most bothersome symptom at baseline (27.3% vs 15.3%, 7.7%, and 5.6% in moderate, good, and improving groups, respectively). Low dose naltrexone was more commonly being taken at baseline among individuals in the severe (45.5%) versus those in the moderate (18%), improving (16.7%), or normal/mild (0%) groups (ASD = 0.649). Other concomitant medications at baseline were similar between PROMIS-PF groups (Supplementary Table 10).

### Trajectory Modeling for Core Severity Measures

We also explored longitudinal trajectory groups for core severity measures ascertained via Likert scales. We identified three longitudinal groups for fatigue, the most common reported symptom at enrollment: one group with what appears to be improving fatigue (*n* = 49), one with what appears to be stable but moderate fatigue (*n* = 76), and one with what appears to be stable and severe fatigue (*n* = 30, Supplementary Figure 9).

Female participants comprised a higher proportion of individuals in the severe fatigue group (70%) compared with those in the moderate (63.2%) or improving (46.9%) groups (ASD = 0.32). The proportion of participants receiving NMV/r was higher in the severe fatigue group (90%) compared with those in the moderate (64.5%) or improving (53.1%) groups (ASD = 0.590). The mean duration between the index infection and randomization was similar in all three groups (Supplementary Table 11), as were concomitant medications (Supplementary Table 12).

Trajectories and groups for other core severity measures (shortness of breath, body aches, cardiovascular symptoms, GI symptoms, and brain fog) are provided in Supplementary Materials (Supplementary Figures 10–13; Supplementary Tables 13–16).

## DISCUSSION

This exploratory analysis of the STOP-PASC clinical trial provides valuable insights into the heterogeneous trajectories of Long COVID symptoms and potential patient-reported outcome measures for their characterization. Using data-driven modeling, we identified distinct trajectories that delineated subgroups of individuals with improving symptoms versus those with persistent or worsening symptoms. We further identified participant features that may be associated with these varied trajectories.

Importantly, we did not identify specific subgroup(s) of individuals who appeared to benefit from NMV/r treatment compared to placebo-ritonavir, although our sample size was limited. Interestingly, groups with worsening PGIC and PROMIS physical function trajectories contained a higher proportion of participants receiving NMV/r compared with those with improving trajectories. These findings align with the primary trial results, as well as a second more recent decentralized clinical trial conducted across the contiguous United States [9], suggesting that a 15-day course of NMV/r does not provide a significant benefit for treating Long COVID in a highly vaccinated population with protracted symptoms. Although a small case series has suggested that some individuals might benefit from extended courses of NMV/r [15], our studies did not identify such subgroups using a data driven approach. In those with protracted Long COVID, antivirals may need to be administered earlier in the illness, before downstream and possibly less reversible adverse effects occur. Indeed, in data from the RECOVER consortium and others, high-risk adult patients with COVID-19 who were treated with Paxlovid within 5 days of SARS-CoV-2 had a lower risk of Long COVID compared to those who did not receive Paxlovid [16–18]. In addition, we did not identify any concomitant medications associated with improving trajectories. Low-dose naltrexone use was more frequent among individuals with worse trajectories. This may reflect confounding by indication, as individuals with greater symptom burden are more likely to receive off-label therapies. Together, these data highlight the need for urgent rigorous testing of targeted therapeutic strategies for different subtypes of Long COVID. This could include (1) enrichment strategies targeting specific subgroups (eg, patients with cardiovascular-predominant symptoms), (2) adaptive trial designs that allow for real-time modification of treatment arms based on emerging trajectory patterns, and (3) stratified randomization by symptom duration and dominant symptom clusters.

Our findings demonstrate that a subset of Long COVID patients experience natural improvement over time, independent of therapeutic intervention. This observation aligns with emerging literature, with ∼50% of patients experiencing spontaneous recovery of fatigue and cognitive deficits months to years after initial infection [19]. However, the extended time interval from initial infection to enrollment (mean 536 days, ∼18 months) indicates that our study predominantly captured chronic rather than subacute Long COVID trajectories. This temporal context may explain why we observed relatively few participants with improving trajectories (11% for PGIS, 32% for fatigue core symptoms), as patients at 18 months post-infection may have developed more entrenched pathophysiological processes less amenable to spontaneous recovery. Although all participants received ritonavir, similar improvement rates between treatment arms suggest that observed recovery patterns likely reflect the natural course of chronic Long COVID rather than drug effects.

The biological mechanisms driving chronic Long COVID may differ substantially from those in the subacute phase (3–6 months post-infection), potentially involving irreversible tissue damage and/or persistent immune dysregulation. Our observation that shorter time since infection was associated with improvement supports this temporal gradient and suggests Long COVID as a biphasic condition requiring different therapeutic approaches at different stages. Chronic Long COVID may therefore require interventions focused on symptom management, rehabilitation, and addressing secondary complications rather than targeting the initial infectious trigger.

Our LCTM modeling revealed additional demographic and clinical differences that may inform pathogenesis and therapeutic strategies. Higher baseline physical function was more common in improving groups, suggesting that preserved functional status at treatment initiation may be associated with more favorable prognosis. Conversely, individuals with cardiovascular symptoms at baseline were more likely to experience worsening trajectories, indicating a potential subgroup that may benefit from targeted interventions aimed at mitigating cardiovascular complications.

Recognizing the critical need for validated Long COVID-specific PRO instruments, several initiatives are underway to address this gap. The NIH RECOVER initiative is conducting comprehensive validation studies of existing PROs for Long COVID use, including the PGIC and PROMIS measures utilized in our study, alongside development of novel Long COVID-specific assessment tools. Beyond RECOVER, international collaborations and academic centers are developing condition-specific instruments that capture the unique features of Long COVID. The validation of such instruments represents a high priority for advancing Long COVID clinical trials, as demonstrated by our findings showing the limitations of repurposed PROs in capturing the nuanced trajectories of this complex condition. Once validated, these Long COVID-specific PROs will enable more precise phenotyping of patient subgroups, better selection of trial endpoints, and improved assessment of therapeutic responses across the heterogeneous spectrum of Long COVID presentations.

This study has limitations. The reliance on data-driven trajectory modeling, while exploratory and hypothesis-generating, introduces subjectivity in the interpretation of clusters and may limit generalizability. Importantly, LCTM clustering solutions are sensitive to the underlying baseline covariate distributions within the study population, meaning that our identified trajectory groups may not emerge identically in cohorts with different demographic compositions or clinical characteristics. Furthermore, the small sample size, particularly in the placebo arm due to the 2:1 randomization scheme, reduced our power. In addition, the limited racial and ethnic diversity of our cohort, with approximately 75% of participants identifying as White, may not be representative of the broader Long COVID population [20] and limits the generalizability of our findings across more diverse populations. Also, the trajectories may not represent pure natural history of Long COVID as ritonavir was given in both groups and some participants were on concomitant medications.

Our findings contribute to the growing understanding of Long COVID's complexity and heterogeneity. By identifying clinical features associated with distinct symptom trajectories, this exploratory analysis provides a foundation for further definition of illness trajectories and prognosis, personalized therapeutic approaches, and improved clinical trial designs. Further validation of PROs tailored to Long COVID and exploration of mechanistic pathways, such as hypercoagulability and ongoing immune activation, will be critical for advancing our ability to address this debilitating condition effectively.

## Supplementary Material

ofaf634_Supplementary_Data

## Supplementary Data

Supplementary materials are available at *Open Forum Infectious Diseases* online. Consisting of data provided by the authors to benefit the reader, the posted materials are not copyedited and are the sole responsibility of the authors, so questions or comments should be addressed to the corresponding author.
