# Supplementary material for: Longitudinal Patient-Reported Outcome Trajectories in Long COVID: Findings From the STOP-PASC Clinical Trial
Source: Open Forum Infect Dis. 2025 Oct 8;12(10):ofaf634. doi: 10.1093/ofid/ofaf634 (PMC12560753; doi:10.1093/ofid/ofaf634)
Supplement: ofaf634_Supplementary_Data [file ofaf634_supplementary_data.docx]

Supplement Table of Contents

[Additional methods 1](#_Toc209181808)

[LCTM model performance 2](#_Toc209181809)

[Baseline Characteristics 9](#_Toc209181810)

[Tables of concomitant medications for PGIS and PGIC LCTM groups 10](#_Toc209181811)

[Post-hoc analysis of a composite of PGIC and PGIS Groups 11](#_Toc209181812)

[Trajectory heatmaps/alluvial plots and tables of group characteristics and concomitant medications for PROMIS – Physical Function 13](#_Toc209181813)

[Trajectory heatmaps/alluvial plots and tables of group characteristics and concomitant medications for Fatigue Core Symptom 15](#_Toc209181814)

[Trajectory heatmaps/alluvial plots and tables of group characteristics for other core symptoms 18](#_Toc209181815)

## Additional methods

**Study Population**

Randomization was stratified by the number of moderate or severe core symptoms (2 or 3 vs >3) and vaccination status (completed primary series vs not completed as defined by CDC). The randomization list for each stratum was generated by block randomization with block size randomly selected from 6 and 9.

**Latent class trajectory modelling**

*Choosing random effect structure and number of groups*

For each of the twelve patient-reported outcomes (PGIC, PGIS, four PROMIS measures, and six core symptoms), we fit a series of latent class trajectory models (LCTMs) with varying random effect structures and number of groups. Random effect structures considered are listed below, ordered from least to most complex:

1. No random effects
2. Random intercepts, common variance-covariance matrix across groups
3. Random intercepts, group-specific variance-covariance matrix
4. Random intercepts and slopes, common variance-covariance matrix across groups
5. Random intercepts and slopes, group-specific variance-covariance matrix

The variance-covariance matrix was always unstructured, and the number of groups varied from 1 to 5. For each outcome, the best-fit model was chosen based on having the lowest Bayesian information criteria (BIC) (Supplementary Table 2). In the case of ties, the simpler model was favored. We confirmed the adequacy of our chosen models by examining multiple diagnostic measures and visually inspecting cluster consensus.

*Model adequacy for chosen models*

We assessed model adequacy of the final best-fit models using several diagnostic measures. This included entropy, a global measure of classification uncertainty and homogeneity within groups. Ideally, entropy should be close to 0, and relative entropy should be close to 1. The area under the curve of the empirical CDF summarizes the distribution of consensus indices (a measure of the stability of groups across many bootstrap samples) and runs from 0 to 1, with 1 being the theoretical best.

## LCTM model performance

Supplementary Table 1: Summary of specification and model adequacy measures for each outcome’s best-fit model. The letter labels for the random effect structures correspond to the list of structures found under the “Choosing random effect structure and number of groups” subsection. Some diagnostic measures could not be calculated for models that identified only one group and are therefore not reported.

| Outcome | Random effect structure | Number of groups | Entropy | Relative entropy | BIC | AIC | Area under CDF curve |
| --- | --- | --- | --- | --- | --- | --- | --- |
| PGIC | b | 2 | 36.66 | 0.65 | 1933.09 | 1911.93 | 0.3 |
| PGIS | b | 2 | 20.33 | 0.81 | 1908.7 | 1887.49 | 0.23 |
| PROMIS - physical function | b | 4 | 19.97 | 0.91 | 4269.75 | 4230.35 | 0.48 |
| PROMIS - fatigue | d | 1 | - | - | 4640.1 | 4621.92 | - |
| PROMIS - dyspnea | d | 1 | - | - | 4554.88 | 4536.7 | - |
| PROMIS - cognitive function | d | 1 | - | - | 4723.8 | 4705.61 | - |
| Fatigue | e | 3 | 46.78 | 0.73 | 3554.27 | 3511.66 | 0.6 |
| Shortness of breath | e | 3 | 42.4 | 0.75 | 3303.32 | 3260.71 | 0.64 |
| Brain fog | d | 1 | - | - | 3555.47 | 3537.21 | - |
| Body aches | e | 2 | 16.6 | 0.85 | 3893.06 | 3862.62 | 0.47 |
| Cardiovascular symptoms | e | 2 | 14.48 | 0.87 | 3487.25 | 3456.81 | 0.4 |
| Gastrointestinal symptoms | e | 2 | 20.96 | 0.8 | 4121.14 | 4090.7 | 0.43 |

For best-fit models that identified more than one group, we also report group-specific diagnostics (Supplementary Table 2):

- APPA (average posterior probability assignment): looks at whether individuals are assigned with high probability and the overall average probability of assignment to each group. Should ideally be greater than 0.7.
- OCC (odds of correct classification): the ratio of the odds of a correct classification into each group. Should ideally be greater than 5.
- Mismatch: the difference between the estimated group proportions and the group membership proportions once individuals have been assigned to a group. Should ideally be close to 0.
- Cluster consensus: a measure of the stability of the group across many bootstrap samples. Runs from 0 to 1 and should ideally be closer to 1.

Supplementary Table 2: Group sizes and group-specific model adequacy measures for best-fit models that identified more than one group.

| Outcome | Measure | Group 1 | Group 2 | Group 3 | Group 4 |
| --- | --- | --- | --- | --- | --- |
| PGIC | N | 130 | 22 | - | - |
|  | % | 85.53 | 14.47 | - | - |
|  | APPA | 0.92 | 0.84 | - | - |
|  | OCC | 2.63 | 22.08 | - | - |
|  | Mismatch | 0.05 | -0.05 | - | - |
|  | Consensus | 0.92 | 0.90 | - | - |
| PGIS | N | 17 | 136 | - | - |
|  | % | 11.11 | 88.89 | - | - |
|  | APPA | 0.86 | 0.96 | - | - |
|  | OCC | 41.03 | 3.57 | - | - |
|  | Mismatch | -0.02 | 0.02 | - | - |
|  | Consensus | 0.90 | 0.97 | - | - |
| PROMIS - physical function | N | 11 | 111 | 13 | 18 |
|  | % | 7.19 | 72.55 | 8.50 | 11.76 |
|  | APPA | 0.89 | 0.96 | 0.97 | 0.94 |
|  | OCC | 86.77 | 9.71 | 384.88 | 118.55 |
|  | Mismatch | -0.01 | 0.02 | 0.00 | 0.00 |
|  | Consensus | 0.94 | 0.91 | 0.90 | 0.90 |
| Fatigue | N | 49 | 30 | 76 | - |
|  | % | 31.61 | 19.35 | 49.03 | - |
|  | APPA | 0.90 | 0.86 | 0.87 | - |
|  | OCC | 16.92 | 27.91 | 7.04 | - |
|  | Mismatch | -0.02 | 0.01 | 0.00 | - |
|  | Consensus | 0.84 | 0.97 | 0.61 | - |
| Shortness of breath | N | 36 | 60 | 59 | - |
|  | % | 23.23 | 38.71 | 38.06 | - |
|  | APPA | 0.87 | 0.87 | 0.92 | - |
|  | OCC | 24.74 | 10.24 | 18.92 | - |
|  | Mismatch | 0.02 | -0.01 | -0.01 | - |
|  | Consensus | 0.98 | 0.74 | 0.83 | - |
| Body aches | N | 25 | 130 | - | - |
|  | % | 16.13 | 83.87 | - | - |
|  | APPA | 0.82 | 0.98 | - | - |
|  | OCC | 26.15 | 8.29 | - | - |
|  | Mismatch | 0.01 | -0.01 | - | - |
|  | Consensus | 0.99 | 0.56 | - | - |
| Cardiovascular symptoms | N | 39 | 116 | - | - |
|  | % | 25.16 | 74.84 | - | - |
|  | APPA | 0.90 | 0.98 | - | - |
|  | OCC | 30.16 | 18.21 | - | - |
|  | Mismatch | 0.01 | -0.01 | - | - |
|  | Consensus | 0.97 | 0.86 | - | - |
| Gastrointestinal symptoms | N | 42 | 113 | - | - |
|  | % | 27.10 | 72.90 | - | - |
|  | APPA | 0.88 | 0.97 | - | - |
|  | OCC | 20.22 | 12.15 | - | - |
|  | Mismatch | 0.01 | -0.01 | - | - |
|  | Consensus | 0.99 | 0.83 | - | - |

*Visualizing model selection and cluster consensus*

For each best-fit model that identified more than one group, we visualized the cluster consensus indices (Supplementary Figures 1-8). The color-coded heatmaps visualize the consensus matrices: each cell corresponds to the probability that two patients are grouped together, with red being probability 1 and white being probability 0. We generally see distinct red squares along the diagonal (one for each group identified by the model) and white panels elsewhere.

We also plot histograms of the distribution of consensus indices. The values in the plots are the same as those shown in the consensus matrix heatmap. In this alternative visualization, it is also apparent that most indices are (close to) 0 or 1, meaning that people are either always grouped together or never grouped together.

Supplementary Figure 1: Heatmap of consensus matrix (left) and distribution of consensus indices (right) for groups identified by best-fit model for PGIC.


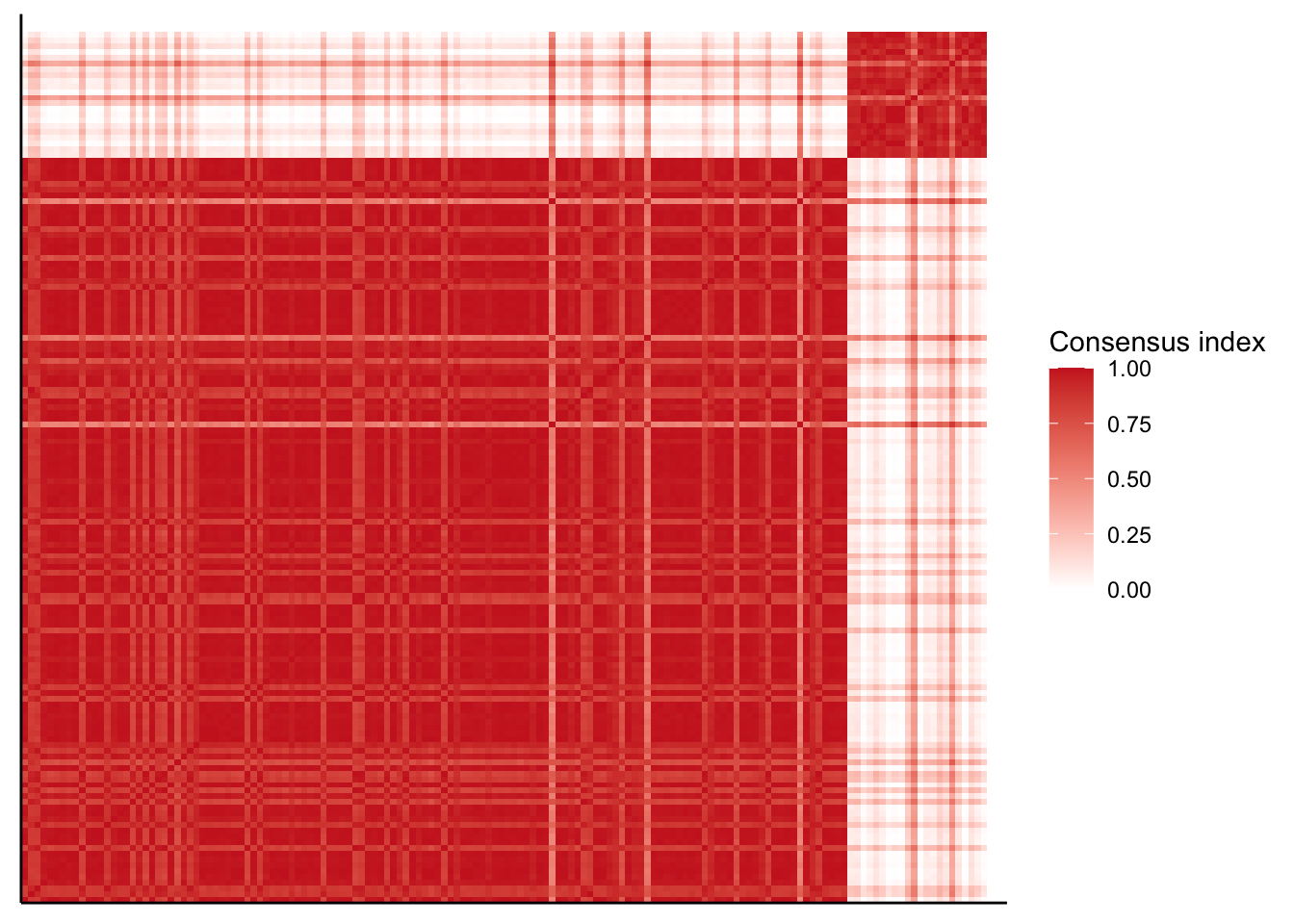

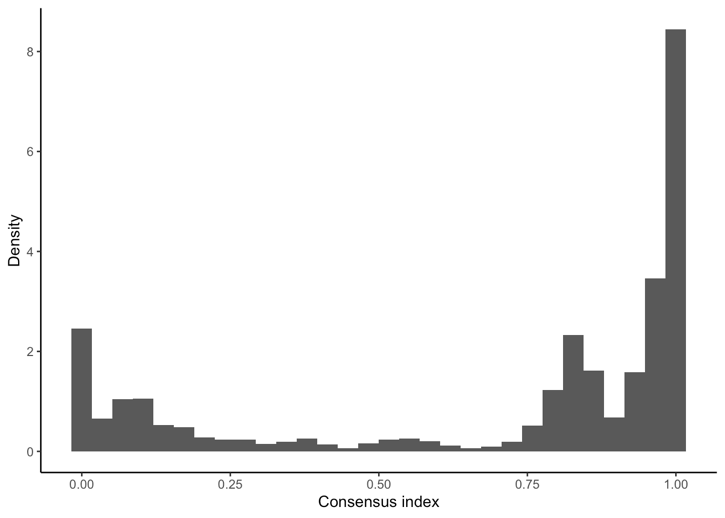


Supplementary Figure 2: Heatmap of consensus matrix (left) and distribution of consensus indices (right) for groups identified by best-fit model for PGIS.


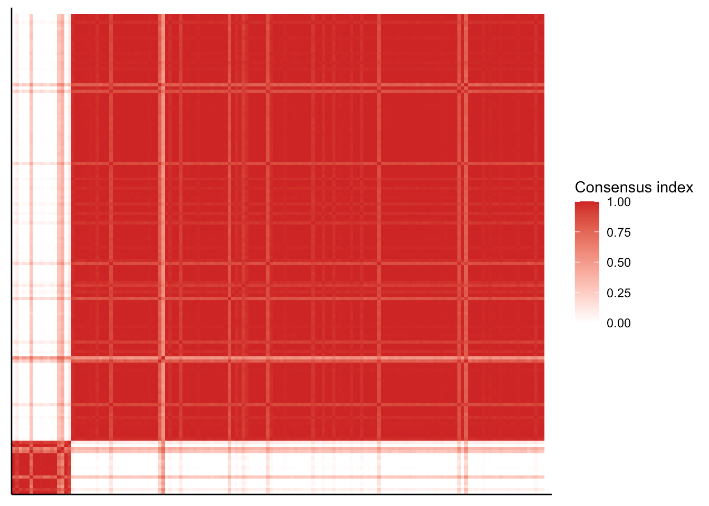

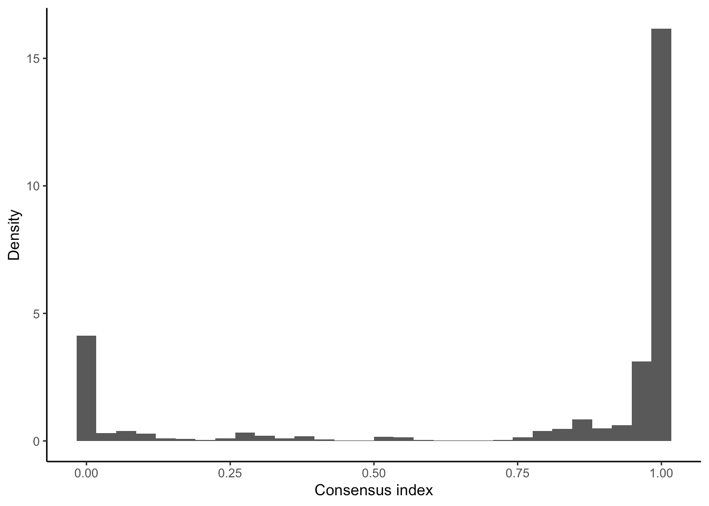


Supplementary Figure 3: Heatmap of consensus matrix (left) and distribution of consensus indices (right) for groups identified by best-fit model for PROMIS - physical function.


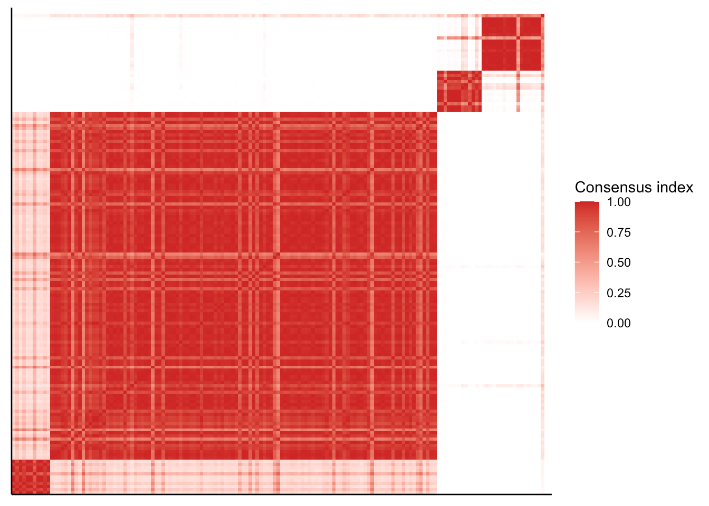

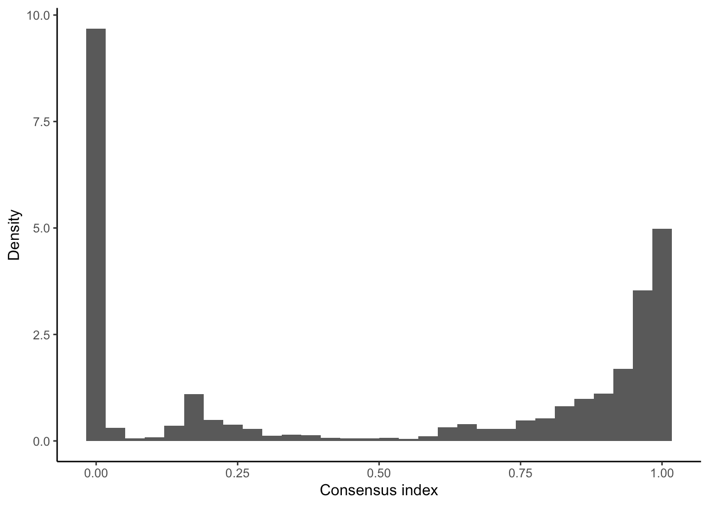


Supplementary Figure 4: Heatmap of consensus matrix (left) and distribution of consensus indices (right) for groups identified by best-fit model for individual fatigue core symptom.


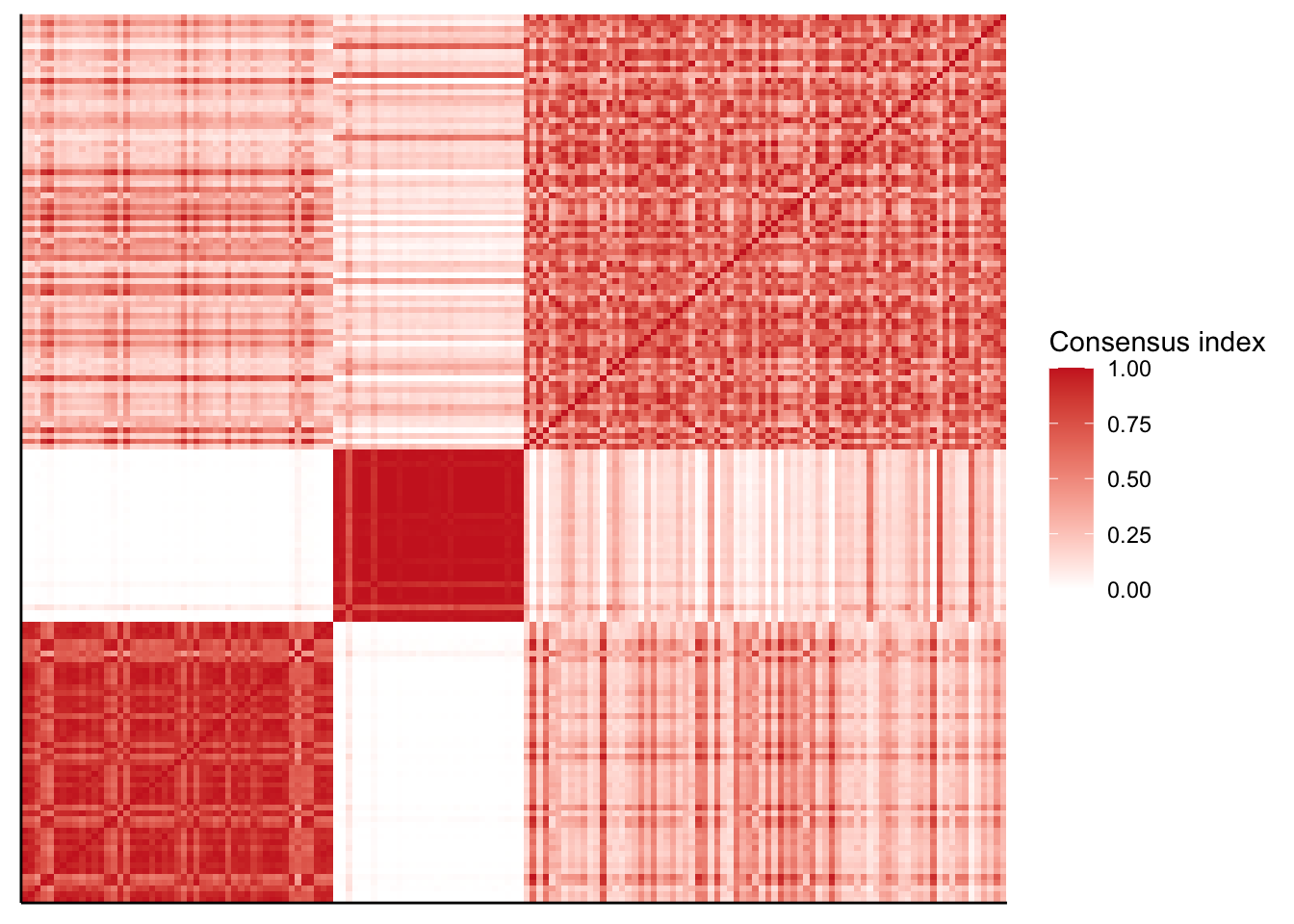

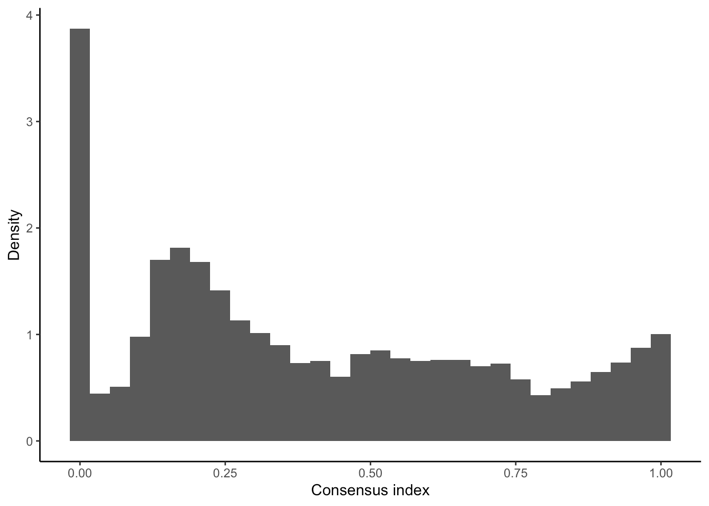


Supplementary Figure 5: Heatmap of consensus matrix (left) and distribution of consensus indices (right) for groups identified by best-fit model for individual shortness of breath core symptom.


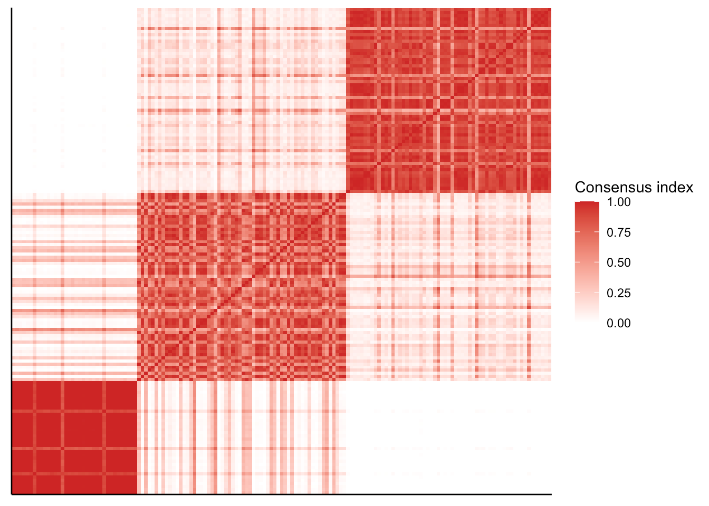

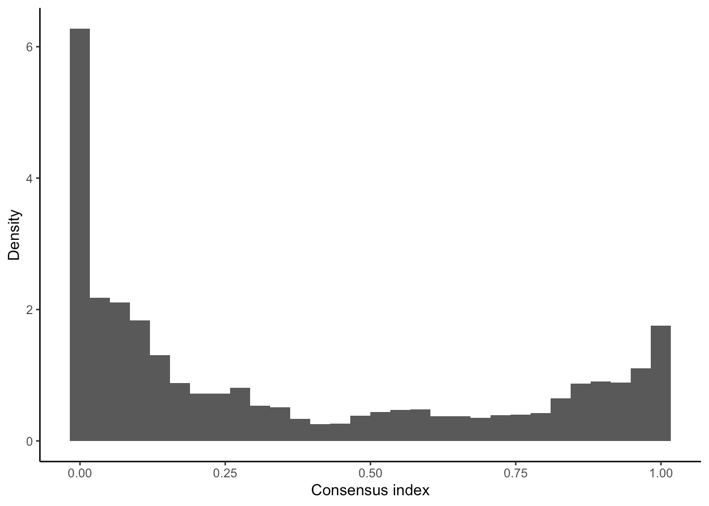


Supplementary Figure 6: Heatmap of consensus matrix (left) and distribution of consensus indices (right) for groups identified by best-fit model for individual body aches core symptom.


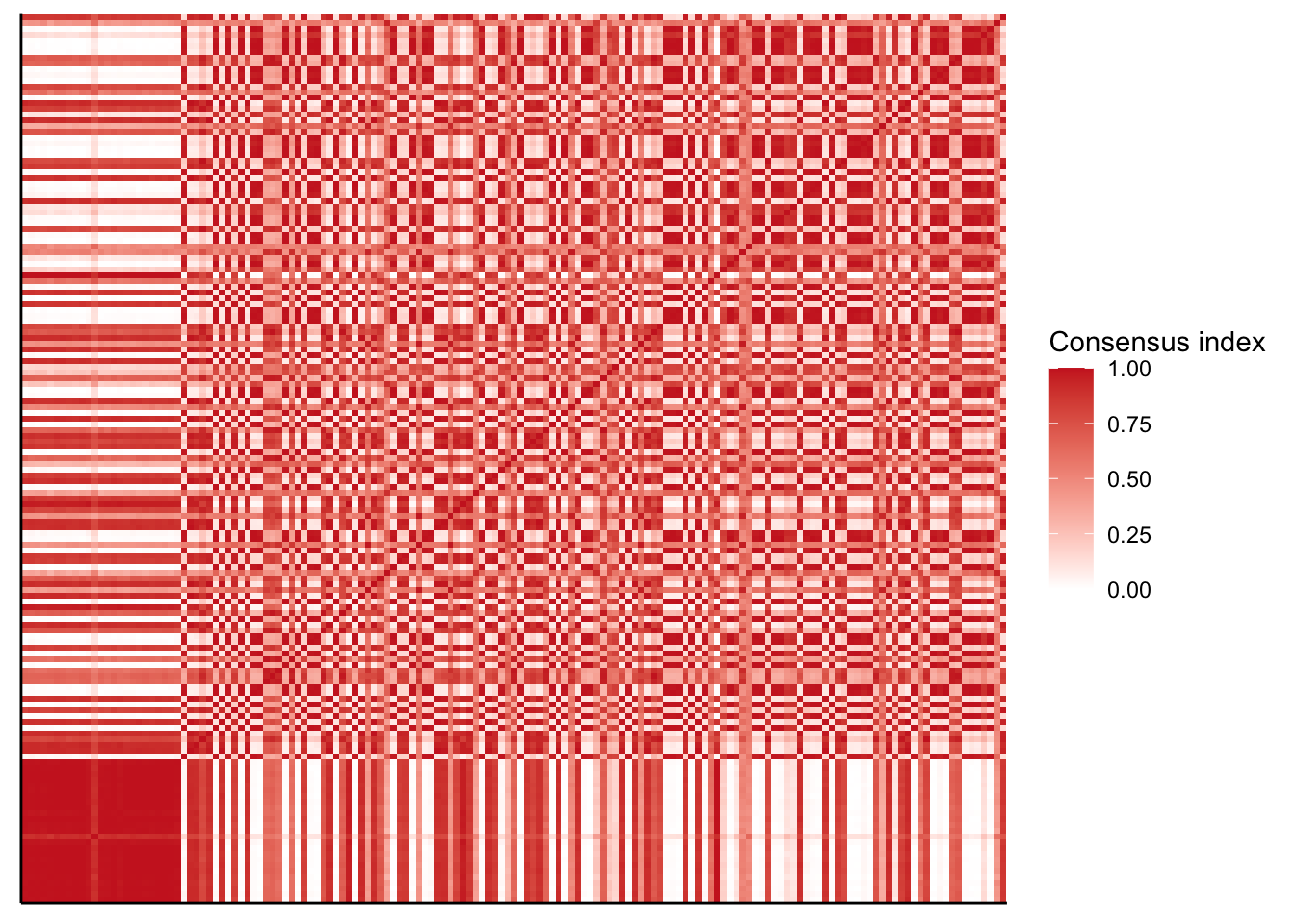

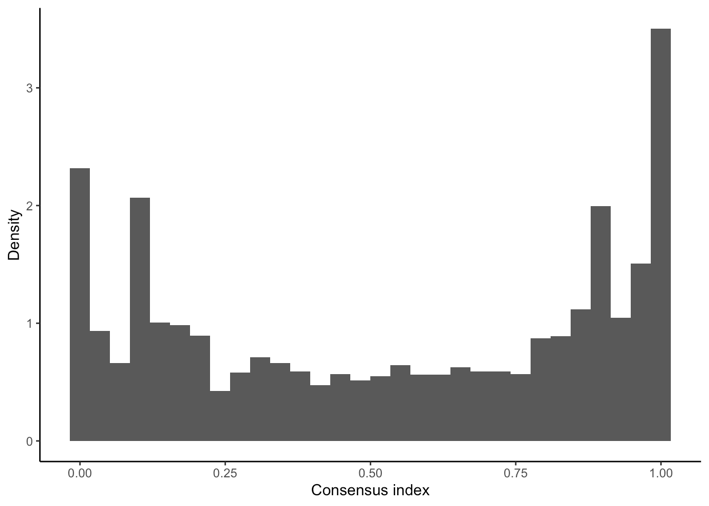


Supplementary Figure 7: Heatmap of consensus matrix (left) and distribution of consensus indices (right) for groups identified by best-fit model for individual cardiovascular core symptoms.


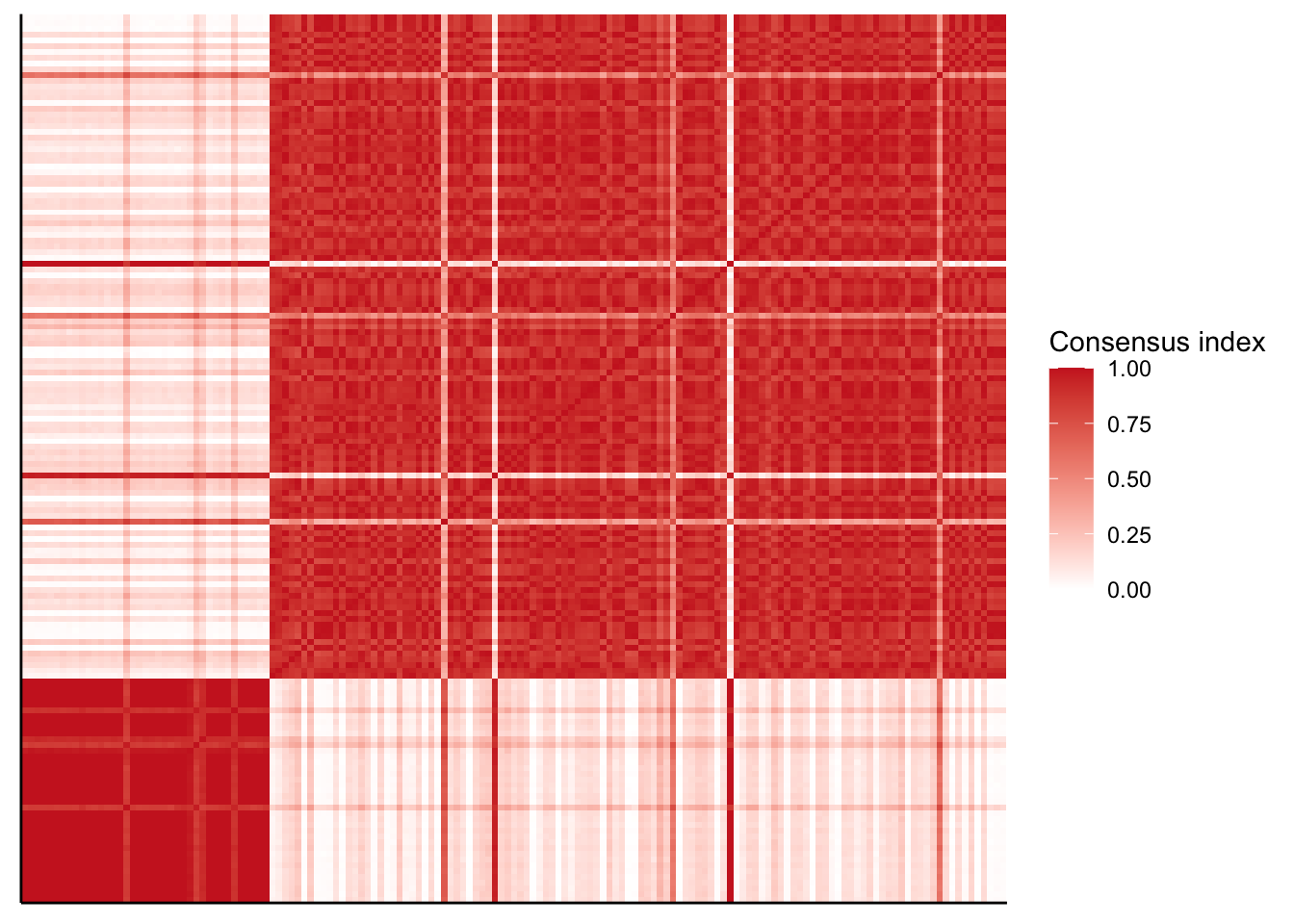

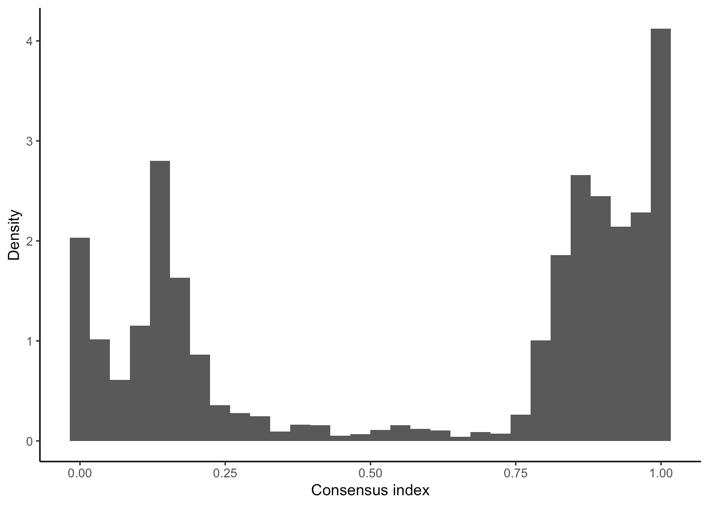


Supplementary Figure 8: Heatmap of consensus matrix (left) and distribution of consensus indices (right) for groups identified by best-fit model for individual gastrointestinal core symptoms.


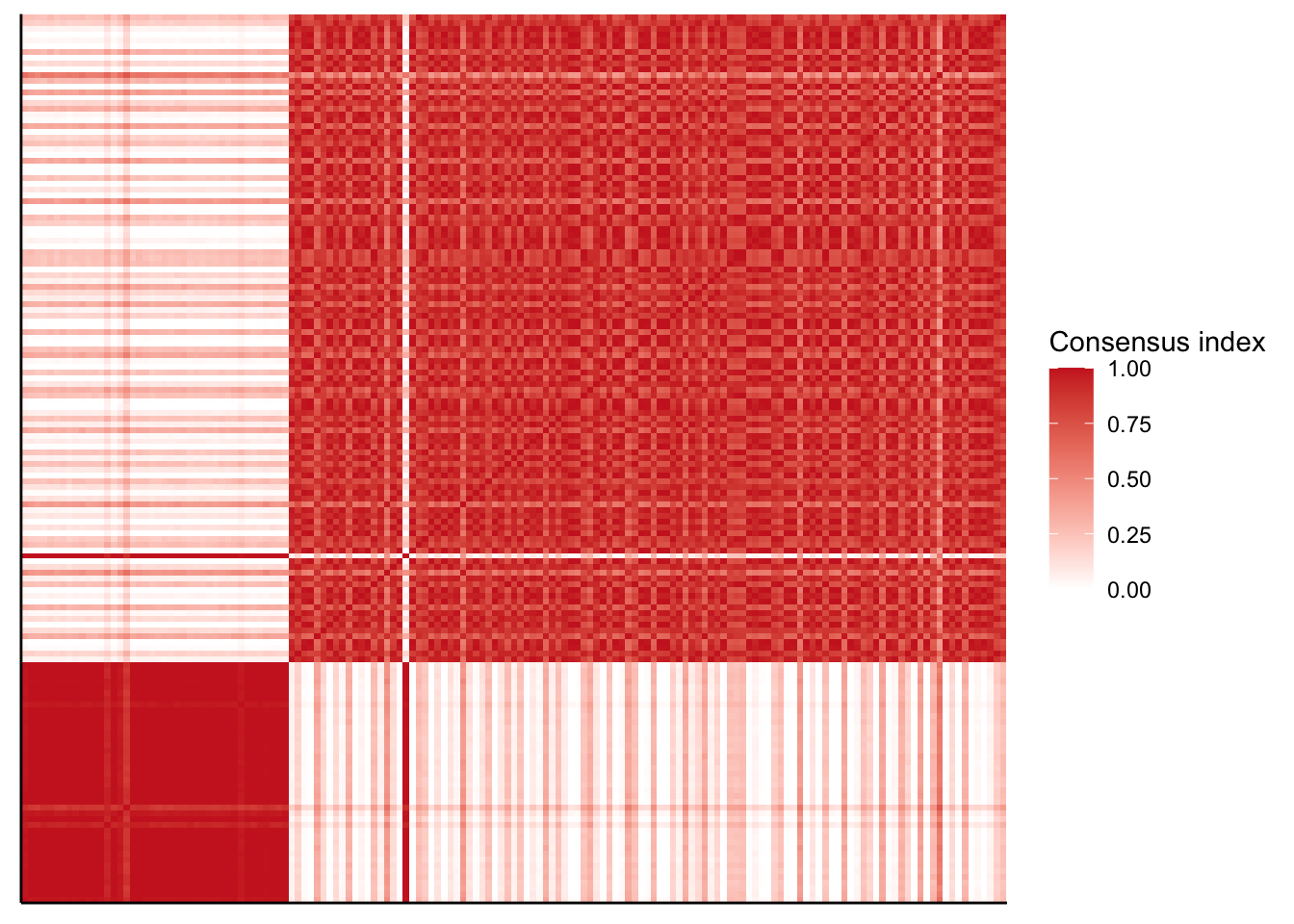

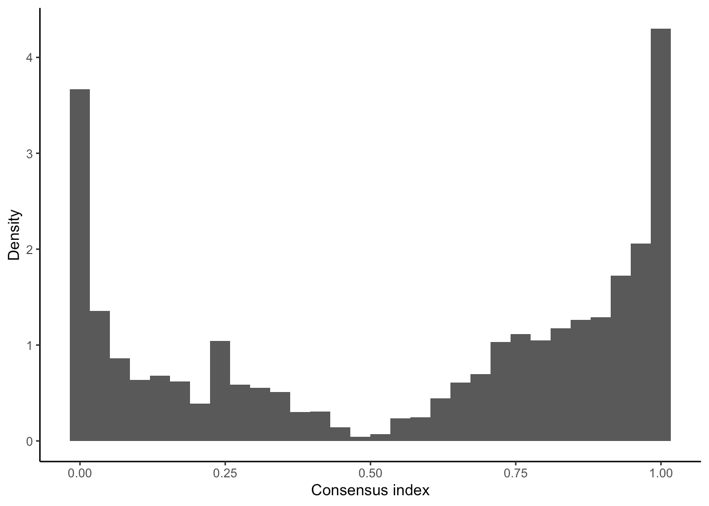


## Baseline Characteristics

Supplementary Table 3: Baseline Characteristics

| **Baseline Characteristic** | **N=155** |
| --- | --- |
| NMV/r arm (%) | 102 (65.8) |
| Female (%) | 92 (59.4) |
| Age (mean (SD)) | 44.25 (13.46) |
| Race (%) |  |
| White | 115 (74.2) |
| Black or African American | 3 (1.9) |
| Asian | 20 (12.9) |
| More than one race | 6 (3.9) |
| Other/Unknown | 11 (7.1) |
| Most bothersome symptom (%) |  |
| Fatigue | 70 (45.2) |
| Shortness of breath | 4 (2.6) |
| Brain fog | 38 (24.5) |
| Body aches | 14 (9.0) |
| Cardiovascular symptoms | 22 (14.2) |
| Gastrointestinal symptoms | 7 (4.5) |
| 1 minute sit-to-stand (mean (SD)) | 20.5 (10.4) |
| Days from index infection to baseline (mean (SD)) | 536 (275) |
| Days from most recent infection to baseline (mean (SD)) | 410 (274) |
| PGIS at baseline (%) |  |
| Not present | 1 (0.7) |
| Very mild | 3 (2.0) |
| Mild | 12 (8.0) |
| Moderate | 86 (57.3) |
| Severe | 40 (26.7) |
| Extremely severe | 8 (5.3) |
| PROMIS Physical function T-Score at baseline (mean (SD)) | 38.31 (7.16) |
| PROMIS Fatigue T-Score at baseline (mean (SD)) | 65.33 (6.48) |
| PROMIS Dyspnea T-Score at baseline (mean (SD)) | 52.32 (7.88) |
| PROMIS Cognitive function T-Score at baseline (mean (SD)) | 36.90 (7.70) |

## Tables of concomitant medications for PGIS and PGIC LCTM groups

Supplementary Table 4: Concomitant medications for the groups identified by PGIS from LCTM

|  | **Group 1 (Improving)**  **(n=17)** | **Group 2 (Worsening)**  **(n=136)** | **ASD** |
| --- | --- | --- | --- |
| Low Dose Naltrexone (Ldn) (Vivitrol, Reviva, Depande, Etc.) | 1 (5.9) | 27 (20) | 0.43 |
| Famotidine (Pepcid, Zantac, Fluxid, Etc.) | 2 (12) | 22 (16) | 0.13 |
| Coenzyme Q10 (CoQ10) | 0 (0) | 8 (5.9) | 0.35 |
| Propranolol (Innopran, Inderal, Hemangeol, Etc.) | 1 (5.9) | 11 (8.1) | 0.09 |
| Cetirizine (Zyrtec) | 3 (18) | 20 (15) | 0.08 |
| Magnesium | 2 (12) | 23 (17) | 0.15 |
| Aspirin | 1 (5.9) | 19 (14) | 0.27 |
| Ivabradine (Corlanor, Procoralan) | 0 (0) | 0 (0) | 0.00 |
| N-Acetylcysteine (NAC) | 1 (5.9) | 3 (2.2) | 0.19 |
| Metoprolol (Lopressor, Toprol XL, Dutoprol, Betaloc, etc.) | 0 (0) | 6 (4.4) | 0.30 |

Supplementary Table 5: Concomitant medications for the groups identified by PGIC from LCTM

|  | **Group 1 (Improving)**  **(n=130)** | **Group 2 (Worsening)**  **(n=22)** | **ASD** |
| --- | --- | --- | --- |
| Low Dose Naltrexone (Ldn) (Vivitrol, Reviva, Depande, Etc.) | 25 (19) | 3 (14) | 0.15 |
| Famotidine (Pepcid, Zantac, Fluxid, Etc.) | 18 (14) | 5 (23) | 0.23 |
| Coenzyme Q10 (CoQ10) | 8 (6.2) | 0 (0) | 0.36 |
| Propranolol (Innopran, Inderal, Hemangeol, Etc.) | 11 (8.5) | 1 (4.5) | 0.16 |
| Cetirizine (Zyrtec) | 19 (15) | 3 (14) | 0.03 |
| Magnesium | 22 (17) | 3 (14) | 0.09 |
| Aspirin | 13 (10) | 7 (32) | 0.56 |
| Ivabradine (Corlanor, Procoralan) | 0 (0) | 0 (0) | 0.00 |
| N-Acetylcysteine (NAC) | 4 (3.1) | 0 (0) | 0.25 |
| Metoprolol (Lopressor, Toprol XL, Dutoprol, Betaloc, etc.) | 5 (3.8) | 1 (4.5) | 0.03 |

## Post-hoc analysis of a composite of PGIC and PGIS Groups

Supplementary Table 6: Cross-tabulation of PGIC scores at week 10 and change in PGIS between weeks 5 and 10

|  | Change in PGIS between weeks 5 and 10 | | | | | | | |  |
| --- | --- | --- | --- | --- | --- | --- | --- | --- | --- |
|  | -4 | -3 | -2 | -1 | 0 | 1 | 2 | 3 | Total |
| PGIC |  |  |  |  |  |  |  |  |  |
| Very much improved | 0 | 1 | 3 | 3 | 3 | 1 | 0 | 0 | 8 |
| Much improved | 1 | 1 | 8 | 8 | 10 | 7 | 0 | 0 | 27 |
| Minimally improved | 0 | 0 | 4 | 4 | 11 | 27 | 0 | 2 | 46 |
| No change | 0 | 0 | 0 | 0 | 5 | 27 | 3 | 0 | 41 |
| Minimally worse | 0 | 0 | 0 | 0 | 1 | 6 | 0 | 0 | 9 |
| Much worse | 0 | 0 | 0 | 0 | 1 | 3 | 0 | 0 | 6 |
| Very much worse | 0 | 0 | 0 | 0 | 0 | 1 | 0 | 0 | 1 |
| Total | 1 | 2 | 15 | 31 | 72 | 12 | 3 | 2 | 138 |

|  | **Group 1**  **(PGIC Improving x PGIS Improving)** | **Group 2**  **(PGIC Improving x PGIS Severe)** | **Group 3**  **(PGIC Worsening x PGIS Severe)** |
| --- | --- | --- | --- |
| n | 17 | 113 | 22 |
| PGIC Improved (%) | 12 (70.6) | 55 (48.7) | 0 (0.0) |
| PGIS Improved (%) | 17 (100.0) | 42 (37.2) | 4 (18.2) |

Supplementary Table 7: Count and percent of patients within each group who improved (with respect to PGIC and PGIS, separately), defined as never worsening and improving between at least one set of timepoints.

Supplementary Table 8: Characterizing PGIC X PGIS Composite Groups

| **Characteristic** | **Group 1 (PGIC Improving X PGIS Improving, n=17)** | **Group 2 (PGIC Improving X PGIC Severe, n =113)** | **Group 3**  **(PGIC Worsening X PGIC Severe, n=22)** | **ASD** |
| --- | --- | --- | --- | --- |
| Treatment arm = NMV/r arm (%) | 11 (64.7) | 72 (63.7) | 18 (81.8) | 0.28 |
| Female (%) | 8 (47.1) | 75 (66.4) | 7 (31.8) | 0.48 |
| Age (mean (SD)) | 44.65 (16.05) | 43.60 (12.96) | 46.27 (13.66) | 0.13 |
| Race (%) |  |  |  | 0.44 |
| American Indian/Alaska Native | 14 (82.4) | 79 (69.9) | 19 (86.4) |  |
| Asian | 0 ( 0.0) | 3 ( 2.7) | 0 ( 0.0) |  |
| Native Hawaiian or other Pacific Islander | 2 (11.8) | 17 (15.0) | 1 ( 4.5) |  |
| Black or African American | 0 ( 0.0) | 5 ( 4.4) | 1 ( 4.5) |  |
| White | 1 ( 5.9) | 9 ( 8.0) | 1 ( 4.5) |  |
| More than one race | 14 (82.4) | 79 (69.9) | 19 (86.4) |  |
| Unknown | 0 ( 0.0) | 3 ( 2.7) | 0 ( 0.0) |  |
| Most bothersome symptom (%) |  |  |  | 0.7 |
| Fatigue | 9 (52.9) | 51 (45.1) | 9 (40.9) |  |
| Shortness of breath | 0 ( 0.0) | 3 ( 2.7) | 1 ( 4.5) |  |
| Brain fog | 7 (41.2) | 24 (21.2) | 6 (27.3) |  |
| Body aches | 1 ( 5.9) | 10 ( 8.8) | 2 ( 9.1) |  |
| Cardiovascular symptoms | 0 ( 0.0) | 18 (15.9) | 4 (18.2) |  |
| Gastrointestinal symptoms | 0 ( 0.0) | 7 ( 6.2) | 0 ( 0.0) |  |
| Heart rate (supine to standing) (mean (SD)) | 8.53 (8.68) | 6.06 (11.29) | 8.55 (9.70) | 0.16 |
| 1 minute sit-to-stand (mean (SD)) | 26.88 (15.16) | 19.43 (9.33) | 20.36 (9.75) | 0.4 |
| Days from index infection to baseline (mean (SD)) | 372.82 (177.55) | 538.65 (278.56) | 625.00 (284.66) | 0.69 |
| Days from most recent infection to baseline (mean (SD)) | 312.12 (166.48) | 414.84 (275.09) | 449.86 (325.29) | 0.37 |
| ASDs (absolute standardized differences) are reported to assess the magnitude of differences between groups: a larger ASD indicates a larger difference between the groups (e.g. 0.2 = small difference, 0.5 = moderate difference, 0.8 = large difference). | | | | |

## Trajectory heatmaps/alluvial plots and tables of group characteristics and concomitant medications for PROMIS – Physical Function

Supplementary Table 9: Characterizing PROMIS – Physical Function Longitudinal Groups

| **Characteristic** | **Group 1**  **(Severe, n=11)** | **Group 2**  **(Moderate, n=111)** | **Group 3**  **(Normal/mild, n=13)** | **Group 4 (Improving, n=18)** | **ASD** |
| --- | --- | --- | --- | --- | --- |
| Treatment arm = NMV/r arm (%) | 7 (63.6) | 77 (69.4) | 5 (38.5) | 12 (66.7) | 0.33 |
| Female (%) | 8 (72.7) | 70 (63.1) | 4 (30.8) | 9 (50.0) | 0.49 |
| Age (mean (SD)) | 46.82 (15.13) | 43.70 (12.18) | 40.62 (17.04) | 47.83 (16.16) | 0.27 |
| Race (%) |  |  |  |  | 0.57 |
| American Indian/Alaska Native | 0 (0.0) | 0 (0.0) | 0 (0.0) | 0 (0.0) |  |
| Asian | 1 (9.1) | 16 (14.4) | 2 (15.4) | 1 (5.6) |  |
| Native Hawaiian or other Pacific Islander | 0 (0.0) | 1 (0.9) | 0 (0.0) | 0 (0.0) |  |
| Black or African American | 1 (9.1) | 1 (0.9) | 0 (0.0) | 1 (5.6) |  |
| White | 9 (81.8) | 78 (70.3) | 11 (84.6) | 15 (83.3) |  |
| More than one race | 0 (0.0) | 5 (4.5) | 0 (0.0) | 1 (5.6) |  |
| Unknown | 0 (0.0) | 10 (9.0) | 0 (0.0) | 0 (0.0) |  |
| Most bothersome symptom (%) |  |  |  |  | 0.69 |
| Fatigue | 5 (45.5) | 50 (45.0) | 5 (38.5) | 9 (50.0) |  |
| Shortness of breath | 0 (0.0) | 4 (3.6) | 0 (0.0) | 0 (0.0) |  |
| Brain fog | 1 (9.1) | 26 (23.4) | 5 (38.5) | 6 (33.3) |  |
| Body aches | 2 (18.2) | 8 (7.2) | 1 (7.7) | 2 (11.1) |  |
| Cardiovascular symptoms | 3 (27.3) | 17 (15.3) | 1 (7.7) | 1 (5.6) |  |
| Gastrointestinal symptoms | 0 (0.0) | 6 (5.4) | 1 (7.7) | 0 (0.0) |  |
| Heart rate (supine to standing) (mean (SD)) | 5.73 (19.80) | 6.23 (9.92) | 10.31 (10.13) | 6.94 (9.56) | 0.20 |
| 1 minute sit-to-stand (mean (SD)) | 10.64 (8.87) | 19.99 (9.12) | 25.69 (8.06) | 24.67 (14.97) | 0.85 |
| Days from index infection to baseline (mean (SD)) | 464.36 (243.37) | 570.71 (278.92) | 507.08 (310.32) | 373.50 (191.20) | 0.42 |
| Days from most recent infection to baseline (mean (SD)) | 395.18 (260.83) | 427.21 (283.10) | 426.92 (333.78) | 310.00 (163.60) | 0.26 |
| ASDs (absolute standardized differences) are reported to assess the magnitude of differences between groups: a larger ASD indicates a larger difference between the groups (e.g. 0.2 = small difference, 0.5 = moderate difference, 0.8 = large difference). | | | | | |

Supplementary Table 10: Concomitant medications for the groups identified by PROMIS-PF from LCTM

|  | **Group 1 (Severe)**  **(n=11)** | **Group 2 (Moderate)**  **(n=111)** | **Group 3 (Normal/Mild) (n=13)** | **Group 4 (Improving) (n=18)** | **ASD** |
| --- | --- | --- | --- | --- | --- |
| Low Dose Naltrexone (Ldn) (Vivitrol, Reviva, Depande, Etc.) | 5 ( 45.5) | 20 ( 18.0) | 0 ( 0.0) | 3 ( 16.7) | 0.649 |
| Famotidine (Pepcid, Zantac, Fluxid, Etc.) | 2 ( 18.2) | 15 ( 13.5) | 3 ( 23.1) | 4 ( 22.2) | 0.141 |
| Coenzyme Q10 (CoQ10) | 1 ( 9.1) | 7 ( 6.3) | 0 ( 0.0) | 0 ( 0.0) | 0.289 |
| Propranolol (Innopran, Inderal, Hemangeol, Etc.) | 2 ( 18.2) | 9 ( 8.1) | 0 ( 0.0) | 1 ( 5.6) | 0.372 |
| Cetirizine (Zyrtec) | 2 ( 18.2) | 16 ( 14.4) | 2 ( 15.4) | 3 ( 16.7) | 0.057 |
| Magnesium | 0 ( 0.0) | 20 ( 18.0) | 2 ( 15.4) | 3 ( 16.7) | 0.340 |
| Aspirin | 1 ( 9.1) | 17 ( 15.3) | 0 ( 0.0) | 2 ( 11.1) | 0.322 |
| Ivabradine (Corlanor, Procoralan) | 0 ( 0.0) | 0 ( 0.0) | 0 ( 0.0) | 0 ( 0.0) | <0.001 |
| N-Acetylcysteine (NAC) | 0 ( 0.0) | 4 ( 3.6) | 0 ( 0.0) | 0 ( 0.0) | 0.137 |
| Metoprolol (Lopressor, Toprol XL, Dutoprol, Betaloc, etc.) | 0 ( 0.0) | 6 ( 5.4) | 0 ( 0.0) | 0 ( 0.0) | 0.169 |

## Trajectory heatmaps/alluvial plots and tables of group characteristics and concomitant medications for Fatigue Core Symptom

Supplementary Figure 9: Fatigue core symptom trajectories visualized within modeling groups using heatmaps (left) and alluvial plots (right).

|  |
| --- |
| **SFig 9**. Fatigue Core Symptom trajectories visualized within modeling groups using heatmaps (left) and alluvial plots (right). |

Supplementary Table 11: Characterizing Fatigue Core Symptom Longitudinal Groups

|  | **Group 1 (Improving)** | **Group 2 (Severe)** | **Group 3 (Moderate)** | **ASD** |
| --- | --- | --- | --- | --- |
| n | 49 | 30 | 76 |  |
| NMV/r arm (%) | 26 (53.1) | 27 (90.0) | 49 (64.5) | 0.59 |
| Female (%) | 23 (46.9) | 21 (70.0) | 48 (63.2) | 0.32 |
| Age (mean (SD)) | 43.69 (13.70) | 43.97 (13.51) | 44.71 (13.45) | 0.05 |
| Race (%) |  |  |  | 0.40 |
| American Indian/Alaska Native | 0 (0.0) | 0 (0.0) | 0 (0.0) |  |
| Asian | 7 (14.3) | 2 ( 6.7) | 11 (14.5) |  |
| Native Hawaiian or other Pacific Islander | 0 (0.0) | 0 (0.0) | 1 (1.3) |  |
| Black or African American | 2 (4.1) | 1 ( 3.3) | 0 ( 0.0) |  |
| White | 35 (71.4) | 24 (80.0) | 56 (73.7) |  |
| More than one race | 1 (2.0) | 2 ( 6.7) | 3 ( 3.9) |  |
| Unknown | 4 (8.2) | 1 ( 3.3) | 5 ( 6.6) |  |
| Ethnicity = Hispanic (%) | 6 (12.2) | 4 (13.3) | 9 (11.8) | 0.03 |
| Most bothersome symptom (%) |  |  |  | 0.51 |
| Fatigue | 18 (36.7) | 15 (50.0) | 37 (48.7) |  |
| Shortness of breath | 1 (2.0) | 1 ( 3.3) | 2 ( 2.6) |  |
| Brain fog | 16 (32.7) | 4 (13.3) | 18 (23.7) |  |
| Body aches | 5 (10.2) | 4 (13.3) | 5 ( 6.6) |  |
| Cardiovascular symptoms | 5 (10.2) | 6 (20.0) | 11 (14.5) |  |
| Gastrointestinal symptoms | 4 (8.2) | 0 ( 0.0) | 3 ( 3.9) |  |
| Acute COVID-19 medication use = Yes (%) | 14 (28.6) | 9 (30.0) | 18 (23.7) | 0.10 |
| Diabetes = Yes (%) | 1 (2.0) | 1 ( 3.3) | 6 ( 7.9) | 0.18 |
| High blood pressure = Yes (%) | 8 (16.3) | 4 (13.3) | 11 (14.5) | 0.07 |
| High cholesterol = Yes (%) | 6 (12.2) | 3 (10.0) | 12 (15.8) | 0.12 |
| Cancer = Yes (%) | 2 (4.1) | 2 ( 6.7) | 2 ( 2.6) | 0.13 |
| Thyroid problem = Yes (%) | 4 (8.2) | 2 ( 6.7) | 10 (13.2) | 0.15 |
| Asthma = Yes (%) | 11 (22.4) | 6 (20.0) | 21 (27.6) | 0.12 |
| Autoimmune disease = Yes (%) | 0 (0.0) | 4 (13.3) | 9 (11.8) | 0.37 |
| Kidney disease = Yes (%) | 1 (2.0) | 0 ( 0.0) | 1 ( 1.3) | 0.14 |
| Liver disease = Yes (%) | 0 (0.0) | 0 ( 0.0) | 0 ( 0.0) | <0.01 |
| Heart disease = Yes (%) | 0 (0.0) | 1 ( 3.3) | 0 ( 0.0) | 0.18 |
| Other comorbidity = Yes (%) | 1 (2.0) | 0 ( 0.0) | 0 ( 0.0) | 0.14 |
| Heart rate (supine to standing) (mean (SD)) | 7.73 (9.27) | 4.53 (13.97) | 6.93 (10.40) | 0.18 |
| SBP (supine to standing) (mean (SD)) | 1.59 (11.11) | 3.07 (13.64) | 2.80 (12.80) | 0.08 |
| DBP (supine to standing) (mean (SD)) | 7.06 (10.63) | 4.43 (7.92) | 6.59 (8.83) | 0.20 |
| 1 minute sit-to-stand (mean (SD)) | 22.24 (10.60) | 19.63 (11.20) | 19.70 (9.85) | 0.17 |
| Days from index infection to baseline (mean (SD)) | 507.22 (292.07) | 543.53 (253.01) | 550.89 (274.19) | 0.11 |
| Days from most recent infection to baseline (mean (SD)) | 369.41 (265.30) | 414.67 (259.56) | 433.92 (284.58) | 0.16 |
| ASDs (absolute standardized differences) are reported to assess the magnitude of differences between groups: a larger ASD indicates a larger difference between the groups (e.g. 0.2 = small difference, 0.5 = moderate difference, 0.8 = large difference). | | | | |

Supplementary Table 12: Concomitant medications for the groups identified by the core fatigue symptom from LCTM

|  | **Group 1 (Improving)**  **(n=49)** | **Group 2 (Severe)**  **(n=30)** | **Group 3 (Moderate) (n=76)** | **ASD** |
| --- | --- | --- | --- | --- |
| Low Dose Naltrexone (Ldn) (Vivitrol, Reviva, Depande, Etc.) | 6 ( 12.2) | 7 ( 23.3) | 15 ( 19.7) | 0.195 |
| Famotidine (Pepcid, Zantac, Fluxid, Etc.) | 6 ( 12.2) | 5 ( 16.7) | 14 ( 18.4) | 0.115 |
| Coenzyme Q10 (CoQ10) | 4 ( 8.2) | 0 ( 0.0) | 4 ( 5.3) | 0.290 |
| Propranolol (Innopran, Inderal, Hemangeol, Etc.) | 6 ( 12.2) | 4 ( 13.3) | 2 ( 2.6) | 0.269 |
| Cetirizine (Zyrtec) | 7 ( 14.3) | 6 ( 20.0) | 10 ( 13.2) | 0.123 |
| Magnesium | 9 ( 18.4) | 4 ( 13.3) | 12 ( 15.8) | 0.092 |
| Aspirin | 5 ( 10.2) | 2 ( 6.7) | 13 ( 17.1) | 0.219 |
| Ivabradine (Corlanor, Procoralan) | 0 ( 0.0) | 0 ( 0.0) | 0 ( 0.0) | <0.001 |
| N-Acetylcysteine (NAC) | 2 ( 4.1) | 0 ( 0.0) | 2 ( 2.6) | 0.202 |
| Metoprolol (Lopressor, Toprol XL, Dutoprol, Betaloc, etc.) | 5 ( 10.2) | 0 ( 0.0) | 1 ( 1.3) | 0.343 |

## Trajectory heatmaps/alluvial plots and tables of group characteristics for other core symptoms

Supplementary Figure 10: Shortness of breath core symptom trajectories visualized within modeling groups using heatmaps (left) and alluvial plots (right).


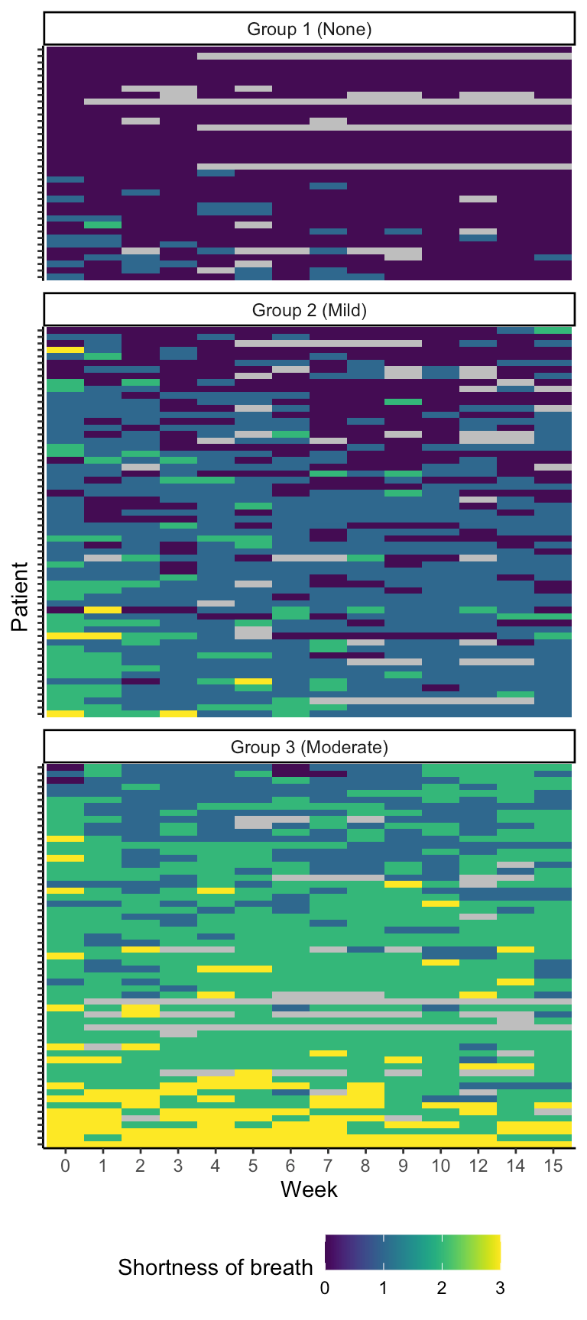

Supplementary Table 13: Characterizing shortness of breath core symptom longitudinal groups. ASDs (absolute standardized differences) are reported to assess the magnitude of differences between groups: a larger ASD indicates a larger difference between the groups (e.g. 0.2 = small difference, 0.5 = moderate difference, 0.8 = large difference).

|  | Group 1 (None) | Group 2 (Mild) | Group 3 (Moderate) | ASD |
| --- | --- | --- | --- | --- |
| n | 36 | 60 | 59 |  |
| NMV/r (%) | 25 (69.4) | 40 (66.7) | 37 (62.7) | 0.09 |
| Female (%) | 18 (50.0) | 36 (60.0) | 38 (64.4) | 0.20 |
| Age (mean (SD)) | 45.83 (15.66) | 43.92 (12.02) | 43.61 (13.57) | 0.10 |
| Race (%) |  |  |  | 0.48 |
| White | 29 (80.6) | 37 (61.7) | 49 (83.1) |  |
| Black or African American | 1 ( 2.8) | 2 ( 3.3) | 0 ( 0.0) |  |
| Asian | 3 ( 8.3) | 9 (15.0) | 8 (13.6) |  |
| More than one race | 1 ( 2.8) | 4 ( 6.7) | 1 ( 1.7) |  |
| Other/Unknown | 2 ( 5.6) | 8 (13.3) | 1 ( 1.7) |  |
| I have complete confidence in this treatment (%) |  |  |  | 0.50 |
| Strongly disagree | 1 ( 2.8) | 1 ( 1.7) | 1 ( 1.7) |  |
| Moderately disagree | 0 ( 0.0) | 0 ( 0.0) | 2 ( 3.4) |  |
| Slightly disagree | 0 ( 0.0) | 3 ( 5.0) | 0 ( 0.0) |  |
| Neither agree nor disagree | 14 (38.9) | 22 (36.7) | 23 (39.0) |  |
| Slightly agree | 5 (13.9) | 4 ( 6.7) | 9 (15.3) |  |
| Moderately agree | 11 (30.6) | 12 (20.0) | 13 (22.0) |  |
| Strongly agree | 5 (13.9) | 18 (30.0) | 11 (18.6) |  |
| Most bothersome symptom (%) |  |  |  | 0.48 |
| Fatigue | 18 (50.0) | 26 (43.3) | 26 (44.1) |  |
| Shortness of breath | 0 ( 0.0) | 1 ( 1.7) | 3 ( 5.1) |  |
| Brain fog | 10 (27.8) | 15 (25.0) | 13 (22.0) |  |
| Body aches | 4 (11.1) | 7 (11.7) | 3 ( 5.1) |  |
| Cardiovascular symptoms | 2 ( 5.6) | 7 (11.7) | 13 (22.0) |  |
| Gastrointestinal symptoms | 2 ( 5.6) | 4 ( 6.7) | 1 ( 1.7) |  |
| Heart rate (supine to standing) (mean (SD)) | 7.83 (8.26) | 5.72 (9.13) | 7.07 (13.56) | 0.14 |
| SBP (supine to standing) (mean (SD)) | 2.31 (11.58) | 3.00 (13.91) | 2.03 (11.39) | 0.05 |
| DBP (supine to standing) (mean (SD)) | 8.06 (8.71) | 6.98 (8.03) | 4.59 (10.55) | 0.25 |
| 1 minute sit-to-stand (mean (SD)) | 22.94 (12.87) | 20.83 (10.28) | 18.64 (8.38) | 0.27 |
| Days from index infection to baseline (mean (SD)) | 466.81 (264.98) | 527.10 (267.45) | 586.39 (282.96) | 0.29 |
| Days from most recent infection to baseline (mean (SD)) | 397.56 (270.08) | 359.90 (250.98) | 468.02 (290.86) | 0.26 |

Supplementary Figure 11: Body aches core symptom trajectories visualized within modeling groups using heatmaps (left) and alluvial plots (right).


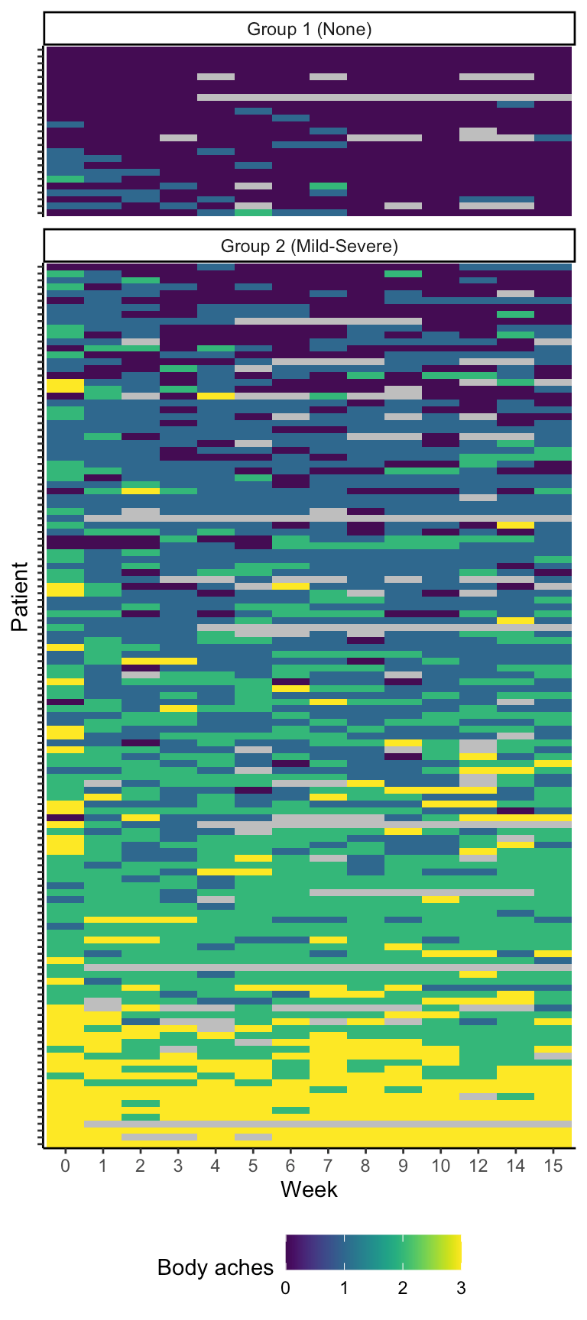

Supplementary Table 14: Characterizing body aches core symptom longitudinal groups. ASDs (absolute standardized differences) are reported to assess the magnitude of differences between groups: a larger ASD indicates a larger difference between the groups (e.g. 0.2 = small difference, 0.5 = moderate difference, 0.8 = large difference).

|  | Group 1 (None) | Group 2 (Mild-Severe) | ASD |
| --- | --- | --- | --- |
| n | 25 | 130 |  |
| NMV/r (%) | 16 (64.0) | 86 (66.2) | 0.05 |
| Female (%) | 9 (36.0) | 83 (63.8) | 0.58 |
| Age (mean (SD)) | 41.84 (14.05) | 44.71 (13.35) | 0.21 |
| Race (%) |  |  | 0.32 |
| White | 17 (68.0) | 98 (75.4) |  |
| Black or African American | 0 ( 0.0) | 3 ( 2.3) |  |
| Asian | 4 (16.0) | 16 (12.3) |  |
| More than one race | 1 ( 4.0) | 5 ( 3.8) |  |
| Other/Unknown | 3 (12.0) | 8 ( 6.2) |  |
| I have complete confidence in this treatment (%) |  |  | 0.41 |
| Strongly disagree | 1 ( 4.0) | 2 ( 1.5) |  |
| Moderately disagree | 0 ( 0.0) | 2 ( 1.5) |  |
| Slightly disagree | 1 ( 4.0) | 2 ( 1.5) |  |
| Neither agree nor disagree | 12 (48.0) | 47 (36.2) |  |
| Slightly agree | 2 ( 8.0) | 16 (12.3) |  |
| Moderately agree | 5 (20.0) | 31 (23.8) |  |
| Strongly agree | 4 (16.0) | 30 (23.1) |  |
| Most bothersome symptom (%) |  |  | 0.76 |
| Fatigue | 12 (48.0) | 58 (44.6) |  |
| Shortness of breath | 0 ( 0.0) | 4 ( 3.1) |  |
| Brain fog | 10 (40.0) | 28 (21.5) |  |
| Body aches | 0 ( 0.0) | 14 (10.8) |  |
| Cardiovascular symptoms | 3 (12.0) | 19 (14.6) |  |
| Gastrointestinal symptoms | 0 ( 0.0) | 7 ( 5.4) |  |
| Heart rate (supine to standing) (mean (SD)) | 8.32 (8.66) | 6.42 (11.21) | 0.19 |
| SBP (supine to standing) (mean (SD)) | 0.64 (12.78) | 2.82 (12.34) | 0.17 |
| DBP (supine to standing) (mean (SD)) | 7.36 (8.95) | 6.12 (9.35) | 0.14 |
| 1 minute sit-to-stand (mean (SD)) | 26.52 (14.74) | 19.33 (8.90) | 0.59 |
| Days from index infection to baseline (mean (SD)) | 373.44 (220.66) | 566.86 (274.15) | 0.78 |
| Days from most recent infection to baseline (mean (SD)) | 320.28 (208.53) | 427.02 (281.86) | 0.43 |

Supplementary Figure 12: Cardiovascular core symptoms trajectories visualized within modeling groups using heatmaps (left) and alluvial plots (right).


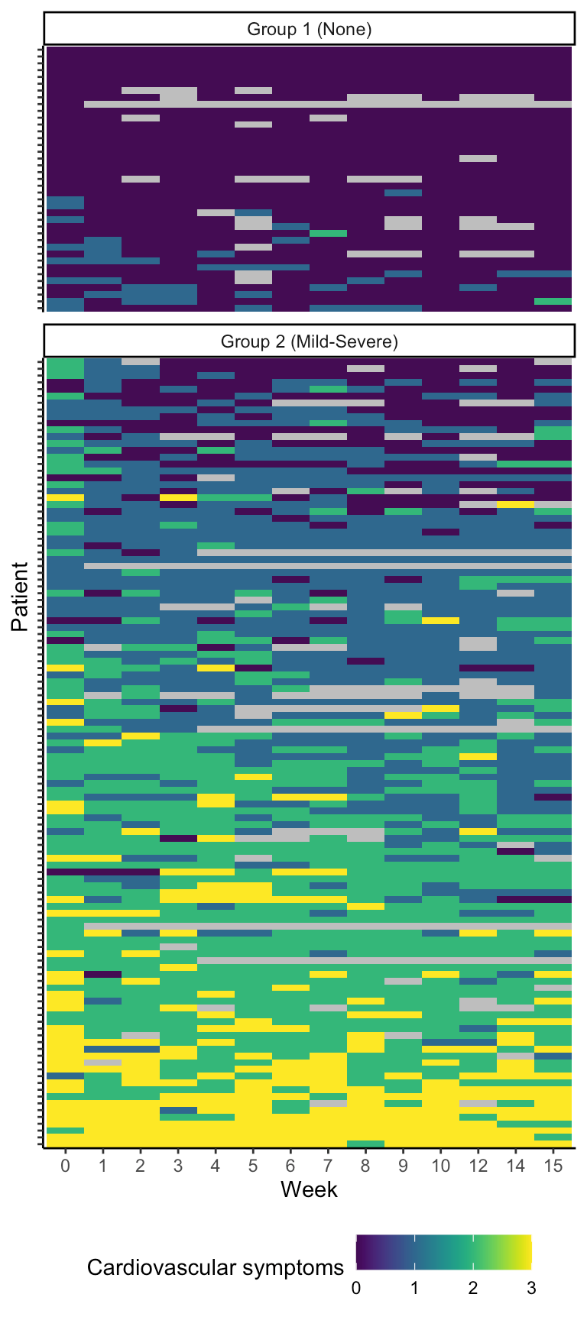

Supplementary Table 15: Characterizing cardiovascular core symptoms longitudinal groups. ASDs (absolute standardized differences) are reported to assess the magnitude of differences between groups: a larger ASD indicates a larger difference between the groups (e.g. 0.2 = small difference, 0.5 = moderate difference, 0.8 = large difference).

|  | Group 1 (None) | Group 2 (Mild-Severe) | ASD |
| --- | --- | --- | --- |
| n | 39 | 116 |  |
| NMV/r (%) | 26 (66.7) | 76 (65.5) | 0.02 |
| Female (%) | 18 (46.2) | 74 (63.8) | 0.36 |
| Age (mean (SD)) | 46.51 (14.35) | 43.48 (13.13) | 0.22 |
| Race (%) |  |  | 0.29 |
| White | 29 (74.4) | 86 (74.1) |  |
| Black or African American | 1 ( 2.6) | 2 ( 1.7) |  |
| Asian | 6 (15.4) | 14 (12.1) |  |
| More than one race | 2 ( 5.1) | 4 ( 3.4) |  |
| Other/Unknown | 1 ( 2.6) | 10 ( 8.6) |  |
| I have complete confidence in this treatment (%) |  |  | 0.41 |
| Strongly disagree | 0 ( 0.0) | 3 ( 2.6) |  |
| Moderately disagree | 0 ( 0.0) | 2 ( 1.7) |  |
| Slightly disagree | 0 ( 0.0) | 3 ( 2.6) |  |
| Neither agree nor disagree | 15 (38.5) | 44 (37.9) |  |
| Slightly agree | 6 (15.4) | 12 (10.3) |  |
| Moderately agree | 9 (23.1) | 27 (23.3) |  |
| Strongly agree | 9 (23.1) | 25 (21.6) |  |
| Most bothersome symptom (%) |  |  | 0.78 |
| Fatigue | 19 (48.7) | 51 (44.0) |  |
| Shortness of breath | 0 ( 0.0) | 4 ( 3.4) |  |
| Brain fog | 13 (33.3) | 25 (21.6) |  |
| Body aches | 5 (12.8) | 9 ( 7.8) |  |
| Cardiovascular symptoms | 0 ( 0.0) | 22 (19.0) |  |
| Gastrointestinal symptoms | 2 ( 5.1) | 5 ( 4.3) |  |
| Heart rate (supine to standing) (mean (SD)) | 8.49 (8.35) | 6.13 (11.53) | 0.23 |
| SBP (supine to standing) (mean (SD)) | -1.44 (11.32) | 3.78 (12.51) | 0.44 |
| DBP (supine to standing) (mean (SD)) | 5.95 (7.81) | 6.45 (9.74) | 0.06 |
| 1 minute sit-to-stand (mean (SD)) | 21.15 (7.72) | 20.27 (11.13) | 0.09 |
| Days from index infection to baseline (mean (SD)) | 487.74 (283.62) | 551.78 (271.43) | 0.23 |
| Days from most recent infection to baseline (mean (SD)) | 430.13 (272.69) | 402.97 (274.79) | 0.10 |

Supplementary Figure 13: Gastrointestinal core symptoms trajectories visualized within modeling groups using heatmaps (left) and alluvial plots (right).


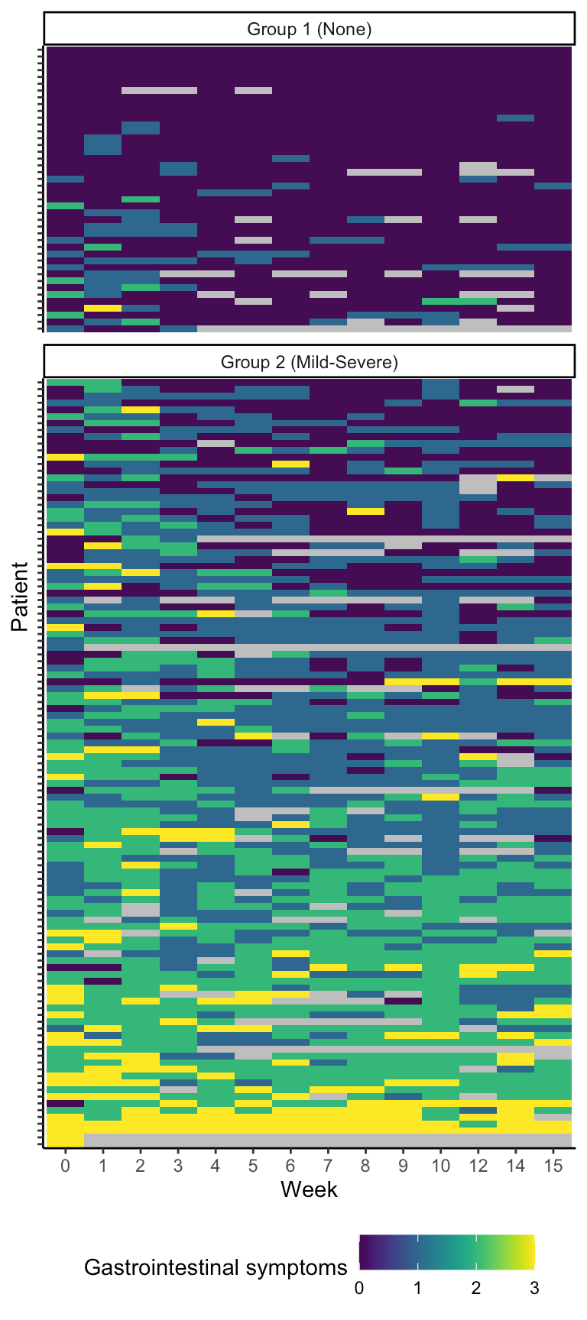

Supplementary Table 16: Characterizing gastrointestinal core symptoms longitudinal groups. ASDs (absolute standardized differences) are reported to assess the magnitude of differences between groups: a larger ASD indicates a larger difference between the groups (e.g. 0.2 = small difference, 0.5 = moderate difference, 0.8 = large difference).

|  | Group 1 (None) | Group 2 (Mild-Severe) | ASD |
| --- | --- | --- | --- |
| n | 42 | 113 |  |
| NMV/r (%) | 26 (61.9) | 76 (67.3) | 0.11 |
| Female (%) | 18 (42.9) | 74 (65.5) | 0.47 |
| Age (mean (SD)) | 45.36 (13.29) | 43.83 (13.56) | 0.11 |
| Race (%) |  |  | 0.39 |
| White | 26 (61.9) | 89 (78.8) |  |
| Black or African American | 1 ( 2.4) | 2 ( 1.8) |  |
| Asian | 9 (21.4) | 11 ( 9.7) |  |
| More than one race | 2 ( 4.8) | 4 ( 3.5) |  |
| Other/Unknown | 4 ( 9.5) | 7 ( 6.2) |  |
| I have complete confidence in this treatment (%) |  |  | 0.20 |
| Strongly disagree | 1 ( 2.4) | 2 ( 1.8) |  |
| Moderately disagree | 0 ( 0.0) | 2 ( 1.8) |  |
| Slightly disagree | 1 ( 2.4) | 2 ( 1.8) |  |
| Neither agree nor disagree | 16 (38.1) | 43 (38.1) |  |
| Slightly agree | 5 (11.9) | 13 (11.5) |  |
| Moderately agree | 10 (23.8) | 26 (23.0) |  |
| Strongly agree | 9 (21.4) | 25 (22.1) |  |
| Most bothersome symptom (%) |  |  | 0.53 |
| Fatigue | 16 (38.1) | 54 (47.8) |  |
| Shortness of breath | 2 ( 4.8) | 2 ( 1.8) |  |
| Brain fog | 15 (35.7) | 23 (20.4) |  |
| Body aches | 4 ( 9.5) | 10 ( 8.8) |  |
| Cardiovascular symptoms | 5 (11.9) | 17 (15.0) |  |
| Gastrointestinal symptoms | 0 ( 0.0) | 7 ( 6.2) |  |
| Heart rate (supine to standing) (mean (SD)) | 7.98 (6.67) | 6.26 (12.02) | 0.18 |
| SBP (supine to standing) (mean (SD)) | 1.29 (14.42) | 2.91 (11.59) | 0.12 |
| DBP (supine to standing) (mean (SD)) | 6.93 (11.03) | 6.10 (8.57) | 0.08 |
| 1 minute sit-to-stand (mean (SD)) | 22.76 (13.21) | 19.65 (9.00) | 0.28 |
| Days from index infection to baseline (mean (SD)) | 462.88 (280.67) | 562.72 (269.16) | 0.36 |
| Days from most recent infection to baseline (mean (SD)) | 411.45 (272.11) | 409.19 (275.40) | 0.01 |
